# Identifying Inpatient Pediatric Services Across National Datasets

**DOI:** 10.1001/jamanetworkopen.2025.13527

**Published:** 2025-06-03

**Authors:** Corrie E. McDaniel, Mark Ralston Daniel, Seneca D. Freyleue, Edouard Seryozhenkov, Amit Peled, Harsha Amaravadi, Niharika Malla, JoAnna K. Leyenaar

**Affiliations:** 1 Department of Pediatrics, Division of Hospital Medicine, University of Washington, Seattle; 2 Data Science, University of Washington; Seattle; 3 The Dartmouth Institute for Health Policy and Clinical Practice, Geisel School of Medicine at Dartmouth, Lebanon, New Hampshire; 4 School of Public Health, University of Washington, Seattle; 5 Dartmouth Health Children’s, Lebanon, New Hampshire

## Abstract

**Importance:**

National statistics about regionalization and access to hospitals’ pediatric services have been derived from different datasets with differing sampling frames, sizes, and designs, generating conflicting estimates about pediatric service accessibility.

**Objective:**

To calculate test characteristics for the provision of pediatric hospital-based inpatient services in 3 national datasets and explore models for improving service identification in a merged dataset.

**Design, Setting, and Participants:**

This cross-sectional study analyzed pediatric services in 3114 US hospitals common across the American Hospital Association Annual Survey (AHA), Centers for Medicare & Medicaid Services Provider of Service File (POS), and National Pediatric Readiness Project (NPRP) in 2021. Analysis was conducted June 2024 to March 2025.

**Exposure:**

Provision of 4 pediatric services—newborn, neonatal intensive care, general pediatric inpatient care, and pediatric intensive care.

**Main Outcomes and Measures:**

Test characteristics and model performance were calculated and reported as F1 scores, a machine learning evaluation metric that calculates the harmonic mean of precision and recall within a model, for the provision of services as reported in the AHA and POS relative to the NPRP, this study’s benchmark for pediatric service reporting. Logistic regression, random forest, gradient-boosted trees, and rule-based models were tested to estimate pediatric service provision using a merged dataset.

**Results:**

Of 3114 hospitals, NPRP identified 2742 providing newborn care (88.1%), 1375 with neonatal intensive care (44.2%), 2204 offering general pediatric care (70.8%), and 450 with pediatric intensive care (14.5%). For newborn care, AHA data showed 95.7% agreement with NPRP (F1 = 0.97; 95% CI, 0.96-0.97), while POS showed 89.4% (F1 = 0.62; 95% CI, 0.60-0.64). For neonatal intensive care, agreement was 89.8% for AHA (F1 = 0.86; 95% CI, 0.85-0.88) and 72.9% for POS (F1 = 0.75; 95% CI, 0.74-0.77). General pediatric care showed lower agreement, with AHA showing 65.6% agreement (F1 = 0.69; 95% CI, 0.67-0.71) and POS showing 69.7% agreement (F1 = 0.79; 95% CI, 0.77-0.80). For pediatric intensive care, AHA agreement was 81.5% (F1 = 0.91; 95% CI, 0.90-0.93) while POS was 78.3% (F1 = 0.49; 95% CI, 0.46-0.51). Merging datasets modestly improved service identification accuracy.

**Conclusions and Relevance:**

In this cross-sectional study of commonly used datasets, reporting of pediatric service provision varied significantly. As these datasets inform pediatric health care policy, these results may guide approaches to optimize service line definitions.

## Key Points

**Question:**

How do 3 commonly cited hospital-level datasets describe the provision of pediatric services differently?

**Findings:**

In this cross-sectional analysis data on 3114 hospitals from of 3 national databases, the American Hospital Association’s annual hospital survey, Centers for Medicare and Medicaid’s Provider of Service file, and the National Pediatric Readiness Project survey had significant variability in descriptions and reporting of pediatric services.

**Meaning:**

These findings suggest that variables and approaches used to describe hospital-based pediatric services using national datasets vary in their accuracy and the corresponding statistical inference regarding pediatric service availability.

## Teaser

This cross-sectional study uses data from the American Hospital Association, the Centers for Medicare & Medicaid Services, and the National Pediatric Readiness Project to examine the level of agreement on hospitals' provision of pediatric services across datasets.

## Introduction

Regionalization of acute, inpatient care for children within the US continues to capture national attention.^1,2^ While regionalization improves outcomes in areas, such as trauma and intensive care, the increasing consolidation of pediatric services has also increased interfacility transfers, travel distances, and disparities in access to inpatient care for general pediatric conditions.^3,4,5,6^ Understanding the availability and accessibility of pediatric services within hospitals is crucial for identifying the drivers and consequences of ongoing regionalization.

National statistics about hospital-level provision of pediatric services often rely on analysis of single datasets.^7,8,9,10,11,12,13,14^ However, datasets vary in sampling frames, sizes, and designs, resulting in variation in findings.^7,13,14^ Without validation of pediatric services as reported within national datasets, there is potential for misclassification of services, overestimation, and underestimation of service availability, and varied conclusions about hospital-level pediatric care.

Thus, we aimed to evaluate (1) the agreement between 3 national datasets in their reporting of 4 pediatric inpatient service lines (newborn, neonatal intensive care, general pediatrics, and pediatric intensive care), and (2) the precision and test characteristics of data reported associated with a pediatric benchmark. As an exploratory aim, we sought to develop models to improve service identification within a merged dataset.

## Methods

### Data Sources

We performed a cross-sectional analysis of services provided in US hospitals in 2021 across 3 data sources. This study follows the Strengthening the Reporting of Observational Studies in Epidemiology (STROBE) reporting guideline. This study was determined by Seattle Children’s institutional review board to be nonhuman participant research.

The first data source was the American Hospital Association’s Annual Survey of Hospitals (AHA).^15^ The AHA data are collected annually through a voluntary survey of more than 6200 hospitals.^15^ The survey is distributed to hospital chief executive officers. Variables include information on pediatric units, pediatric bed counts, service lines, hospital system affiliations, and physician staffing.

The second data source was the Centers for Medicare & Medicaid (CMS) Provider of Service file (POS).^16^ The POS is publicly available and collected quarterly from all institutions that receive Medicare funding. The POS consists of data collected by the CMS state survey agency and hospital designees for the application and maintenance of Medicare and Medicaid eligibility. The hospital and nonhospital facilities files provide information on hospital certification, termination, accreditation, and types of services provided.^16^ As the raw data represents all active providers, there are more than 150 000 entities described quarterly within the POS.

The third data source was the National Pediatric Readiness Program (NPRP) survey.^12^ The NPRP is a pediatric-specific assessment administered through the Emergency Medical Services for Children Data Center (EMS-C) conducted in 2013 and 2021. The development and administration of the NPRP survey is supported by the Health Resources and Services Administration, EMS-C, and cosponsored by the American Academy of Pediatrics, American College of Emergency Physicians, and the Emergency Nurses Association.^17,18,19,20^ The assessment was completed by an individual with specific clinical knowledge of pediatric care at each institution, often an emergency department nurse manager. A total of 5150 emergency departments were contacted in 2021, excluding Veteran Affairs and prison hospitals. The survey includes hospital classifications, pediatric services, and information on board certification of physicians.^12^ Given the pediatric specific focus of this survey, state-level data quality verification,^12^ and its extensive use for pediatric health services research,^12,21,22,23,24,25^ we used the NPRP as the benchmark for provision of pediatric services.

### Identification of Pediatric Services

**Figure 1.**
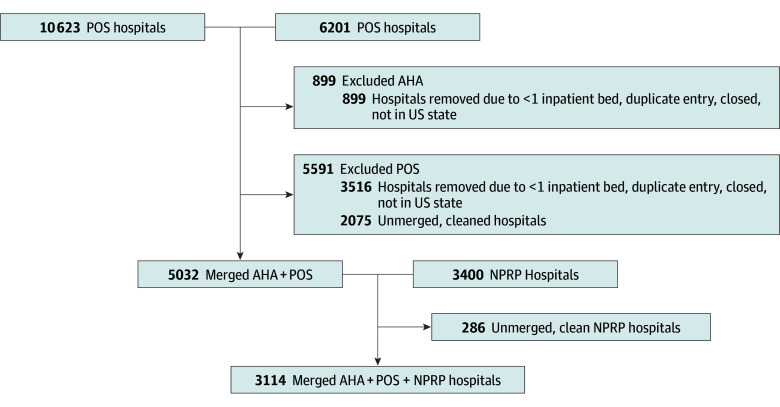
Flow Diagram for Merging the American Hospital Association (AHA) Annual Hospital Survey, Centers for Medicare and Medicaid Services Provider of Service (POS) File, and National Pediatric Readiness Program (NPRP) Survey for 2021

To identify the presence of pediatric services within a hospital, we merged the 3 data sources across a common year, 2021. Within each dataset we limited to acute care hospitals in US states (excluding territories). Full data processing steps for merging across datasets are available in eTable 1 in Supplement 1. We analyzed the same set of acute care hospitals represented across each of the 3 datasets (N = 3114 hospitals) (Figure 1).

Within each dataset, we identified 4 pediatric service lines: (1) newborn, (2) neonatal intensive care, (3) general pediatric inpatient care, (4) pediatric intensive care (eTable 2 in Supplement 1). Variables available to identify service lines varied between datasets (Table 1). For example, to define the provision of newborn care, respondents to the NPRP reported if their hospital provided on-site provision of newborn nursery. However, the AHA and POS do not define newborn care directly, rather the provision of newborn care is based upon the provision of obstetric care as a proxy. For example, AHA defines newborn care as a statement of provision of obstetric (OB) services, 1 or more OB bed, or level 1 or higher OB care; and POS defines newborn care by a statement of provision of OB services. Both the AHA and POS separate neonatal intensive care from neonatal intermediate care, while the NPRP does not. For this work, we defined neonatal intensive care to include both neonatal intermediate and intensive-level care.

**Table 1.** Variables Describing Pediatric Services Within a Shared Set of 3114 Hospitals Within the National Pediatric Readiness Project (NPRP), American Hospital Association (AHA) Annual Survey, and the Centers for Medicare and Medicaid Services Provider of Service (POS) Datasets

Service	NPRP^a^		AHA^b^		POS^c^	
	Dataset variable	No. (%)	Definition^d^	No. (%)	Definition^d^	No. (%)
Newborn care	On-site provision of newborn care^e^	1872 (60)	Provision of obstetric care	1535 (49)	Obstetric care	2080 (67)
			≥1 Obstetric bed	1515 (49)		
			≥Level 1 OB care	1500 (48)		
			Provision of obstetric care or ≥1 obstetric bed or ≥ level 1 OB care	1535 (49)		
Neonatal care	On-site provision of neonatal intensive care	880 (28)	Provision of neonatal-intermediate care	438 (14)	Neonatal intermediate care	1509 (48)
			≥1 Neonatal intermediate care bed	340 (11)		
			Provision neonatal-intensive care	665 (20)	Neonatal intensive care	708 (23)
			≥1 Neonatal-intensive care bed	638 (20)		
			Provision of neonatal-intermediate care or ≥1 neonatal intermediate care bed or provision neonatal-intensive care or ≥1 neonatal-intensive care bed	867 (28)	Neonatal intermediate or neonatal-intensive care	1545 (50)
General pediatric care	On-site provision of pediatric care within a designated pediatric inpatient ward	991 (32)	Provision of pediatric medical-surgical care	1052 (34)	General pediatric care	2182 (70)
	On-site provision of pediatric care within an adult inpatient ward	1511 (49)	≥1 Pediatric medical-surgical care bed	752 (24)		
	On-site provision of pediatric care within a designated pediatric inpatient ward or within an adult inpatient ward	2090 (67)	Provision of pediatric medical-surgical care or ≥1 pediatric medical-surgical care bed	1052 (34)		
Pediatric intensive care	On-site provision of pediatric intensive care within a designated pediatric intensive care unit	288 (9)	Provision of pediatric intensive care	259 (8)	Pediatric intensive care	421 (14)
	On-site provision of pediatric intensive care within an adult intensive care unit	608 (20)	≥1 Pediatric intensive care bed	233 (7)		
	On-site provision of pediatric intensive care within a designated pediatric intensive care unit or within an adult intensive care unit	805 (26)	Provision of pediatric intensive care or ≥1 pediatric intensive care bed	259(8)		

^a^ Missing variables within the NPRP: newborn, 18 (0.6%); neonatal intensive care 27 (0.9%); general inpatient pediatric care, 4 (0.1%); pediatric intensive care, 12 (0.4%).

^b^ Missing variables within the AHA Annual Survey: 796 (25.6%) across all domains of pediatric services.

^c^ Missing variables within the POS file through Centers for Medicare and Medicaid Services: 26 (0.8%).

^d^ If a hospital provides obstetric services; they must also care for normal newborns. Provision of obstetric services was used as a proxy for nursery services.

^e^ For each of the columns, the numerator is the number of hospitals defined by each variable and the denominator is the number of hospitals identifiable within each dataset.

### Statistical Analysis

#### Dataset Agreement

We examined counts and proportions of hospitals reporting each service line within each dataset. To compare datasets, we defined the provision of service inclusively (favoring sensitivity over specificity), defining presence of service as the positive response on any identified variable associated with that service line. For example, for general inpatient pediatric care, if a hospital responded on the NPRP that they had on-site provision of pediatric inpatient care on a pediatric ward or if they provided pediatric care on an adult inpatient ward, we said that hospital had general pediatrics services. Then, we plotted the intersections of provision of services as a matrix, using UpSet diagrams to visualize the distribution of agreement for each service-line between datasets.^26^

#### Evaluation of Test Characteristics and Precision Comparing AHA and POS With the NPRP

We then sought to compare definitions of service line provision as reported in the AHA and POS with the NPRP. For newborn and neonatal intensive care, the NPRP included a single variable indicating service availability. For general inpatient pediatric care and pediatric intensive care, the NPRP had multiple variables. Using this dataset, we defined availability of pediatric services in 2 ways: a pediatric-only definition, which identified on-site provision of pediatric care within a designated pediatric inpatient ward and on-site provision of pediatric intensive care within a designated pediatric intensive care unit, and a comprehensive definition, which represented on-site provision of pediatric care within a designated pediatric inpatient ward or within an adult inpatient ward and on-site provision of pediatric intensive care within a designated pediatric intensive care unit or within an adult intensive care unit.

We assessed test characteristics (ie, sensitivity, specificity, positive estimative value [PEV] and negative estimative value [NEV], and likelihood ratios) with 95% CIs comparing service line variables for the AHA and the POS with the NPRP. We examined F1 scores—a classification evaluation metric that calculates the harmonic mean of precision and recall of a model—for each combination of definitions to identify the combination of variables with the highest performance between the AHA or POS and the NPRP.^27^

For these calculations, we limited the data to hospitals that reported all the variables across a given service line for consistency in the denominator. We evaluated all single variables (eg, “AHA OB beds ≥1”) and 2 or more variables in combination with ANDs or ORs between variables (eg, “AHA OB services AND OB beds ≥1 AND OB level ≥1”). We limited the analysis to logical combinations of ANDs and ORs of the variables, as decided by the research team. However, as a sensitivity analysis, we examined all potential combinations of AHA variables. Procedures for missing data, bias assessment, and detailed explanation of comparisons available in eAppendix 1 in Supplement 1.

#### Exploratory Models for Merged Datasets

Lastly, we used logistic regression, 2 decision tree analyses—random forest analysis (RF)^28^ and gradient-boosted trees analysis (XGBoost)^29^—and rule-based sequential reasoning to estimate hospital services when using a combined AHA and POS dataset, using the NPRP as the pediatric benchmark. Exploratory variables included geographic designation (county, state, and census region), all pediatric service-line and pediatric psychiatry variables, hospital bed counts, and facility characteristics as defined in the AHA and POS (eAppendix 2 in Supplement 1). Detailed descriptions of methods for model development are available in eAppendix 3 and eTables 3 and 4 in Supplement 1. We calculated test characteristics for each model using the combination dataset. Analyses were conducted June 2024 to March 2025 using Python versions 3.12 and 3.10.9 (Python Software Foundation) with scikit-learn, xgboost, upsetplot, numpy, pandas, and matplotlib packages.

## Results

In 2021, 3114 hospitals were present across all 3 datasets. The POS contained 5032 unique acute care hospitals (97.0% response rate), the AHA contained 4995 hospitals (71.0% response rate), and the NPRP had 3644 (response rate of 78.7%). Within the cleaned, premerged datasets, in the AHA 1450 of 4995 hospitals (29.0%) hospitals were missing all responses to questions about pediatric service-line characteristics, 289 hospitals (2.7%) in the POS were missing all service line data, and no hospitals in the NPRP were missing all service line data.

### Definitions and Agreement

**Figure 2.**
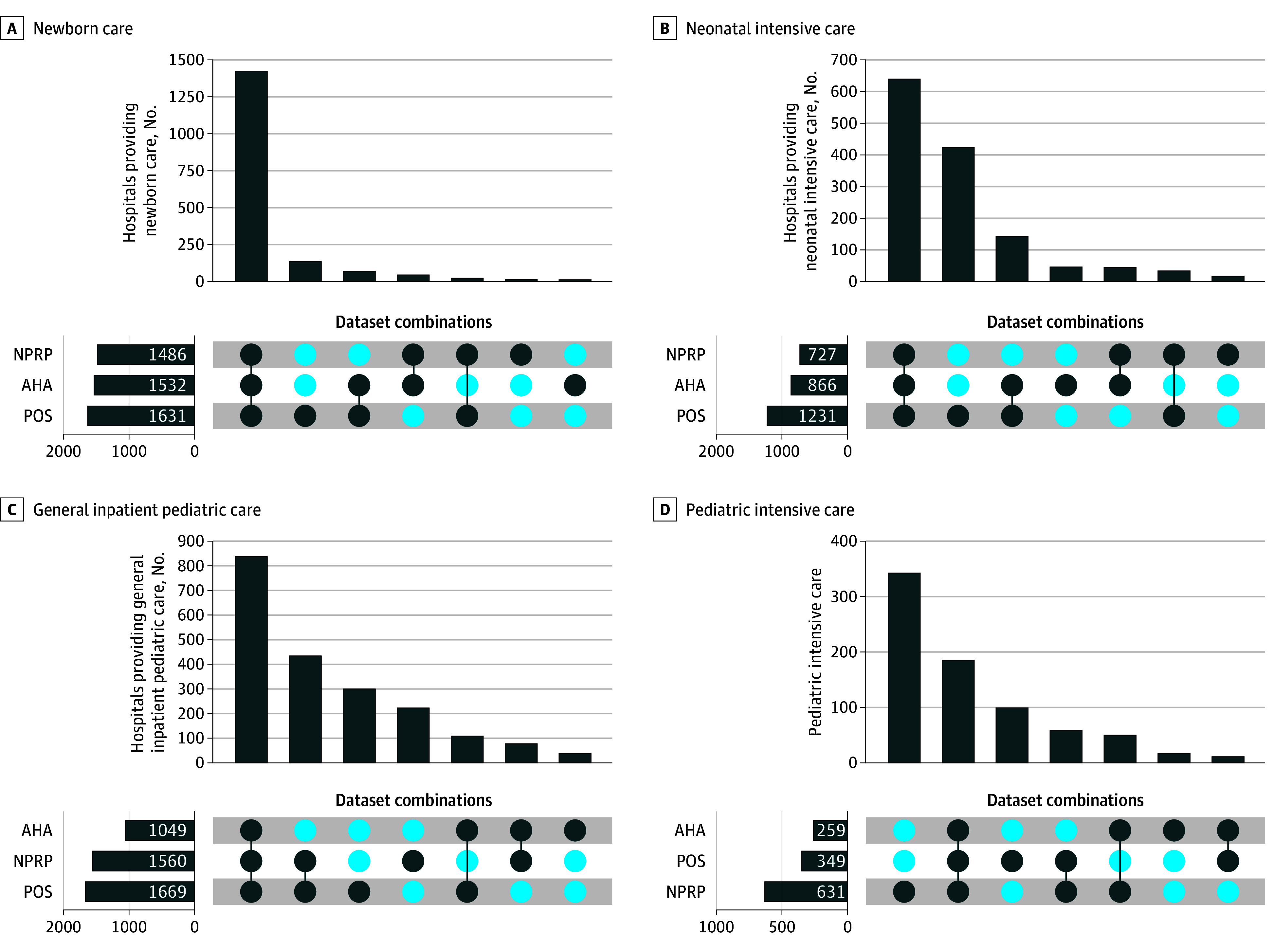
Agreement in Provision of Services Between the American Hospital Association (AHA) Annual Hospital Survey, Centers for Medicare and Medicaid Services Provider of Service (POS) File, and National Pediatric Readiness Program (NPRP) Survey Datasets for 2021 Based on Inclusive Definitions The horizontal bars on the left represent the total number of hospitals identified by each individual data source as providing the specified service. The vertical bars display the number of hospitals identified by each unique combination of data sources. The dot matrix below the vertical bars shows which data source(s) contributed to each intersection: dark blue dots indicate inclusion in the intersection, while light blue dots indicate exclusion. Dark blue bars represent the size of each intersection group.

We observed wide variation in the reported availability of pediatric services across datasets (Table 1). The highest level of agreement across datasets for neonatal intensive and pediatric intensive care was among hospitals reporting that they did not have services (957 of 3114 [30.7%] and 1535 [49.3%] hospitals, respectively). Datasets agreed that 613 hospitals (19.7%) did not provide newborn care and 303 (10.0%) did not provide general pediatrics care. When looking at the provision of services, newborn care had the highest level agreement (AHA had 95.7% agreement with the NPRP; POS had 89.4% agreement), while hospitals with general pediatric care had the lowest (Figure 2). For pediatric intensive care, the NPRP identified 341 hospitals (11.0%) not identified by AHA or POS. Except for pediatric intensive care, the POS identified more hospitals with a given service than the AHA or the NPRP.

### Test Characteristics

Table 2 shows test characteristics for AHA and POS variables when compared with the NPRP, including the inclusive definition of service lines and the highest performing variables. Complete reporting of variable combinations is in eTable 5 in Supplement 1.

**Table 2.** Test Characteristics and Accuracy for the AHA’s Annual Hospital Survey and CMS’ Provider of Service File Variable Combinations for Provision of Pediatric Services Using the NPRP as the Benchmark for Provision of Pediatric Services

Characteristic	Sensitivity (95% CI)	Specificity (95% CI)	PEV (95% CI)	NEV (95% CI)	LR+ (95% CI)	LR− (95% CI)	F1 (95% CI)^a^
**Newborn care, n = 2317 hospitals**							
NPRP definition: onsite provision of newborn nursery							
AHA							
Inclusive definition provision of obstetric services or obstetric level ≥1 or obstetric beds ≥1	0.98 (0.98-0.99)	0.91 (0.89-0.93)	0.95 (0.94-0.96)	0.97 (0.95-0.98)	10.89 8.77-13.51)	0.02 (0.01-0.03)	0.97 (0.96-0.97)
Highest performance provision of obstetric services or obstetric level ≥1^b^	0.98 (0.98-0.99)	0.91 (0.89-0.93)	0.95 (0.94-0.96)	0.97 (0.95-0.98)	10.89 (8.77-13.51)	0.02 (0.01-0.03)	0.97 (0.96-0.97)
POS							
Highest performance provision of obstetric services^c^	0.97 (0.96-0.98)	0.76 (0.73-0.79)	0.88 (0.86-0.89)	0.93 (0.91-0.95)	4.02 (3.56-4.53)	0.04 (0.03-0.06)	0.92 (0.91-0.93)
**Neonatal intensive care, n = 2317 hospitals**							
NPRP definition: onsite provision of neonatal intensive care							
AHA							
Inclusive definition provision of neonatal intensive care services or NICU beds ≥1 or neonatal intermediate services or neonatal intermediate beds ≥1	0.94 (0.92-0.95)	0.88 (0.87,0.90)	0.78 (0.76-0.81)	0.97 (0.96-0.98)	8.0 (6.94-9.11)	0.07 (0.06-0.10)	0.85 (0.84-0.87)
Highest Performance NICU beds ≥1 or neonatal intermediate care beds ≥1	0.92 (0.90-0.94)	0.91 (0.89-0.92)	0.82 (0.79-0.84)	0.96 (0.95-0.97)	9.79 (8.39-11.43)	0.01 (0.07-0.12)	0.86 (0.85-0.88)
POS							
Inclusive definition provision of neonatal intensive care services or intermediate care services	0.89 (0.87-0.92)	0.64 (0.62-0.67)	0.54 (0.51-0.56)	0.93 (0.91-0.94)	2.52 (2.34-2.70)	0.17 (0.13-0.21)	0.67 (0.65-0.69)
Highest performance provision of neonatal intensive care services	0.68 (0.65-0.71)	0.94 (0.93-0.95)	0.84 (0.81-0.87)	0.87 (0.85-0.88)	11.64 (9.50-14.7)	0.34 (0.30-0.38)	0.75 (0.74-0.77)
**General pediatric inpatient care, n = 2206 hospitals**							
Pediatric-only NPRP definition: onsite provision of inpatient pediatric care in a pediatric ward							
AHA							
Inclusive definition general inpatient pediatric care serves or general pediatric care beds ≥1	0.84 (0.84-0.86)	0.74 (0.72-0.77)	0.62 (0.59-0.65)	0.90 (0.88-0.92)	3.26 (2.97-3.58)	0.22 (0.19-0.26)	0.71 (0.69-0.73)
Highest performance general pediatric care beds ≥1	0.74 (0.71-0.78)	0.89 (0.88-0.91)	0.78 (0.75-0.81)	0.87 (0.86-0.89)	7.0 (5.97-8.11)	0.29 (0.25-0.33)	0.76 (0.74-0.78)
POS							
Highest performance: provision of general pediatrics^c^	0.94 (0.92-0.96)	0.38 (0.36-0.41)	0.43 (0.41-0.46)	0.93 (0.91-0.95)	1.53 (1.46-1.60)	0.16 (0.12-0.21)	0.59 (0.57-0.61)
Comprehensive NPRP definition: onsite provision of inpatient pediatric care in a pediatric ward or pediatric care on an adult ward							
AHA							
Inclusive definition general inpatient pediatric care serves or general pediatric care beds ≥1	0.57 (0.55-0.60)	0.82 (0.79-0.85)	0.88 (0.85-0.90)	0.47 (0.44-0.50)	3.19 (2.71-3.77)	0.52 (0.49-0.56)	0.69 (0.67-0.71)
Highest performance general pediatric care beds ≥1	0.57 (0.55-0.60)	0.82 (0.79-0.85)	0.88 (0.85-0.90)	0.47 (0.44-0.50)	3.19 (2.71-3.77)	0.52 (0.49-0.56)	0.69 (0.67-0.71)
POS							
Highest performance: provision of general pediatrics^c^	0.81 (0.79-0.83)	0.46 (0.42-0.50)	0.77 (0.75-0.79)	0.52 (0.48-0.56)	1.50 (1.39-1.61)	0.42 (0.36-0.47)	0.79 (0.77-0.80)
**Pediatric intensive care, n = 1665- hospitals**							
Pediatric-only NPRP definition: onsite provision of pediatric intensive care within a pediatric intensive care unit							
AHA							
Inclusive definition: pediatric intensive care services or PICU beds ≥1	0.93 (0.90-0.97)	0.98 (0.97-0.98)	0.84 (0.79-0.89)	0.99 (0.11-1.00)	38.09 (27.53-52.70)	0.68 (0.04-0.11)	0.88 (0.87-0.90)
Highest performance: PICU beds ≥1	0.91 (0.87-0.95)	0.99 (0.98-0.99)	0.91 (0.87-0.95)	0.99 (0.98-0.99)	78.9 (49.09-126.82)	0.09 (0.06-0.14)	0.91 (0.90-0.93)
POS							
Highest performance provision of pediatric intensive care services^c^	0.80 (0.74-0.85)	0.90 (0.88-0.91)	0.51 (0.45-0.56)	0.97 (0.96-0.98)	7.70 (6.52-9.09)	0.23 (0.17-0.30)	0.62 (0.60-0.64)
Comprehensive NPRP definition: on-site provision of pediatric intensive care within a pediatric intensive care unit or pediatric intensive care in an adult intensive care unit							
AHA							
Inclusive definition: pediatric intensive care services or PICU beds ≥1	0.33 (0.29-0.37)	0.97 (0.97-0.99)	0.90 (0.86-0.94)	0.73 (0.80-0.75)	15.52 (10.20-23.63)	0.68 (0.64-0.72)	0.49 (0.46-0.51)
Highest performance: PICU beds ≥1	0.33 (0.29-0.37)	0.97 (0.97-0.99)	0.90 (0.86-0.94)	0.73 (0.80-0.75)	15.52 (10.20-23.63)	0.68 (0.64-0.72)	0.49 (0.46-0.51)
POS							
Highest performance: provision of pediatric intensive care services^c^	0.36 (0.32-0.40)	0.91 (0.89-0.93)	0.69 (0.64-0.74)	0.72 (0.70-0.74)	4.02 (3.23-5.01)	0.70 (0.66-0.75)	0.47 (0.45-0.50)

Abbreviations: AHA, American Hospital Association; LR−, likelihood ratio negative; LR+, likelihood ratio positive; NEV, negative estimative value; NICU, neonatal intensive care unit; NPRP, National Pediatric Readiness Project; PEV, positive estimative value; PICU, pediatric intensive care unit; POS, Provider of Service File.

^a^ F1 is a machine learning evaluation metric that calculates the harmonic mean of precision and recall within a model. A high F1 score indicates a combination of variables with a high performance to the NPRP.

^b^ AHA variables “OB services,” “OB beds ≥ 1,” “OB level ≥ 1” and “OB services OR OB beds ≥ 1” all generated the same test characteristics. This is because if a hospital indicated they had services beds or services there was then no missing data for OB level.

^c^ For POS service lines that only have a single variable, we report only the highest performance scores, although these are the same as the inclusive definition scores given that there is only 1 variable.

#### Newborn Care

Across 2317 hospitals with no missing data across all 3 datasets, AHA variables for “provision of obstetric services” and “≥1 OB bed” had the highest performance (F1 = 0.97; 95% CI, 0.96-0.97) (Table 2). All AHA definitions for newborn care yielded a sensitivity of more than 0.97 and a specificity of 0.91; the positive estimative value (PEV) and negative estimative values (NEV) were more than 0.95 and all likelihood ratios (LR) more than 10.

Within the POS, the single variable “provision of obstetric services” had an overall performance score of 0.92 (95% CI, 0.91-0.93) with a sensitivity of 0.97 (95% CI, 0.96-0.98), a specificity of 0.76 (95% CI, 0.73-0.79), a PEV of 0.88 (95% CI, 0.85-0.89), and a NEV of 0.04 (95% CI, 0.03-0.06).

#### Neonatal Intensive Care

The combination with the highest performance for AHA variables was “≥1 neonatal intensive OR intermediate care bed," with an F1 score of 0.86 (95% CI, 0.84-0.88) (Table 2). Nine different combinations of AHA variables for neonatal intensive care yielded a sensitivity of more than 90% and specificity of more than 88%, 4 combinations had PEV of more than 0.90, and 13 had NEV of more than 0.90 (eTable 5 in Supplement 1).

For the POS, the combination with the highest performance score was “the neonatal intensive care services alone” (F1 = 0.75; 95% CI, 0.74-0.77) (Table 2). A single combination of POS variables for neonatal intensive care yielded a sensitivity of more than 90% and 2 combinations yielded a specificity of of more than 90%. No combinations had both a sensitivity and specificity of more than 90%.

#### General Inpatient Care

For general inpatient pediatric care services, 2206 hospitals had no missing data. When compared with the pediatric-only NPRP definition (ie, on-site provision of inpatient pediatric care in a pediatric ward), the AHA variable “≥ 1 pediatric bed” had the highest performance (F1 = 0.76; 95% CI, 0.74-0.78) (Table 2). When compared with the comprehensive NPRP definition (ie, onsite provision of inpatient pediatric care in a pediatric ward OR pediatric care on an adult ward), the AHA variable of “≥ 1 pediatric bed” again had the highest performance (F1 = 0.69; 95% CI, 0.67-0.71), although with a lower overall score than compared with the pediatric-only definition. Two combinations within the AHA had sensitivities of more than 80% and 1 different AHA combinations had specificities of more than 90%.

For the POS, when compared with the pediatric-only NPRP definition, the POS variable “provision of general pediatrics” had an overall performance score of F1 of 0.59 (95% CI, 0.57-0.61) (Table 2). When compared with the comprehensive NPRP definition, this POS variable had an overall higher performance (F1 = 0.79; 95% CI, 0.77-0.80). Two combinations had sensitivity of more than 90%; no combination had a specificity of more than 50%.

#### Pediatric Intensive Care

For all pediatric intensive care variables, 1665 hospitals had no missing data. When compared with the pediatric-only NPRP definition, the AHA variable “≥ 1 pediatric intensive care bed” demonstrated the highest performance (F1 = 0.91; 95% CI, 0.90-0.93) (Table 2). When comparing to the comprehensive NPRP definition, the highest performing AHA variable was “≥ 1 pediatric intensive care bed,” although with a lower overall performance than compared with the pediatric-only definition (F1 = 0.49; 95% CI, 0.46-0.51). For the AHA, 8 combinations had a sensitivity of more than 90%, 14 had a specificity of more than 90%, 6 had a PEV of more than 0.9, and 8 had a NEV of more than 0.99.

When compared with the pediatric-only NPRP definition, the POS variable “provision of pediatric intensive care” had a performance score of 0.62 (95% CI, 0.60-0.64). When compared with the comprehensive definition, the performance score dropped to 0.47 (95% CI, 0.45-0.50). No combination of POS variables had a sensitivity of more than 90% and 2 had a specificity of more than 90%.

### Sensitivity Analysis for All Variable Combinations and Exploratory Models for Merged Datasets

Neither the order in rankings by F1 scores nor the test characteristics changed when all combinations of variables within each service line were assessed. The exploratory models performed with relatively high NEV for newborn care (logistic regression: 0.93; 95% CI, 0.90-0.96; to sequential rule-based and XGBoost: 0.97; 95% CI, 0.95-0.99), neonatal intensive care (logistic regression and random forest: 0.94; 95% CI, 0.92-0.96; to sequential rule-based model and XGBoost: 0.95; 95% CI, 0.93-0.97), and pediatric intensive care (random forest and logistic regression: 0.98; 95% CI, 0.97-0.99; to sequential rule-based and XGBoost: 0.99; 95% CI, 0.98-1.00). Models demonstrated less robust NEV for general pediatric care but had higher PEV (sequential rule-based: 0.76; 95% CI, 0.73-0.80; to logistic regression: 0.81; 95% CI, 0.77-0.84). PEV was highest (PEV of more than 0.90) in newborn care across all models and in pediatric intensive care for XGBoost and logistic regression (eTable 6 in Supplement 1).

## Discussion

In this study, commonly used datasets, including the AHA Annual Hospital Survey, the CMS POS, and the NPRP survey, varied significantly in the variables used to report pediatric inpatient services. Combinations of variables to define service lines yielded a range of differing test characteristics associated with the NPRP. Inclusive definitions, where each service line was defined broadly by any variable being present, often yielded low estimative scores. Exploratory models using the AHA and POS datasets in combination generally demonstrated improved sensitivity in estimation of services compared with the use of a single dataset.

Data to assess availability of pediatric services is critical to health service planning, yet we identified significant differences across datasets that make understanding pediatric services nationally challenging. The datasets demonstrated different sampling frames, nonresponse rates, missing data percentages, and variables to define service lines. For example, when using the AHA variable of 1 or more pediatric bed to define general pediatric services, only 24% of hospitals within the AHA identified as having pediatric services. This is different from 70% in the POS. Furthermore, when we assessed performance of variables using the F1 score, an evaluation metric that balances precision as a measure of correctness with recall,^27^ AHA and POS newborn and AHA pediatric intensive care unit variables performed with F1 scores exceeding 0.9 (excellent), while even the highest performing variables for general pediatric care reached F1 scores ranged from 0.59 to 0.79 (fair to adequate).

Published literature reports highly variable estimates of general inpatient pediatric services. Cushings et al^7^ used AHA survey data to assess inpatient pediatric services, defining inpatient units by the presence of designated beds, and they reported a loss of almost 20% of inpatient pediatric units from 2008 to 2018.^7^ Replicating similar methods, Michelson et al^30^ found a decline of approximately 30% of pediatric inpatient units from 2008 to 2022. These findings are different from those by San Soucie et al,^31^ which defined general pediatric inpatient units as first the presence of general pediatric services in the POS or if missing in the POS, then presence of services in the AHA. San Soucie et al^31^ identified a net closure rate of 2% from 2011 to 2018. This 10-fold discrepancy resulting from methodologic differences in datasets and variable definitions creates uncertainty when advocating for policy to support children nationally.

Multiple studies that include patient-level use data demonstrate that a large fraction of general hospitals in the US continue over time to admit patients under the age of 18 years,^32,33,34,35^ although with a similar pattern of contraction.^13,36^ In a study conducted in Illinois, VonAchen et al^13^ assessed unit closure compared with pediatric inpatient days. While they identified a reduction of 26% of licensed pediatric beds in Illinois from 2012 to 2017, a quarter of hospitals that closed pediatric beds continued to admit more than 50% of their previous volume, and fewer than 20% of closures had substantive pediatric volume loss. These studies demonstrate the complexities of identifying pediatric-serving hospitals. First, a large number of hospitals admit small numbers of children.^33,35^ Second, while many hospitals are losing pediatric-designated beds and units, there is nuance in that loss. In rural areas, even small reductions in pediatric capacity may dramatically impact access to care for children.^3^ Conversely, in some hospitals with historically low pediatric admissions, the formal removal of pediatric-designated beds may reflect an administrative change rather than a meaningful reduction in actual care delivery. This variability underscores the importance of context-specific assessment when evaluating changes in pediatric capacity.

The results of this study demonstrate that individual datasets provide inconsistent pictures of pediatric service availability, which has implications for policy development. We caution against using any single dataset in isolation, particularly for cross-sectional analyses. Instead, we recommend using triangulated data from multiple sources when possible and selecting service line definitions that optimize NEVs or PEVs based on the specific research question. For example, using high negative estimative value definitions when identifying hospitals without pediatric services and high positive estimative definitions when confirming service availability.

Beyond these methodologic recommendations, our findings highlight the need for standardized definitions of pediatric services across national hospital surveys and datasets, with clear distinctions between dedicated pediatric-specific inpatient capacity (specialized beds, units, and staff) and the acceptance of children on adult units. The latter approach may be constrained by various factors including age restrictions, overall hospital capacity, nursing expertise and staffing ratios, pediatric-specific equipment availability, and clinician comfort with pediatric care. Establishing consistent definitions and measurement approaches would enable more accurate monitoring of pediatric capacity and capability over time. Furthermore, such standardization would allow for meaningful comparisons of outcomes and resource use between dedicated pediatric settings and mixed adult-pediatric care environments. National pediatric and hospital organizations should collaborate to develop these standardized definitions and advocate for broad implementation.

Future research directions could address the limitations of the current hospital-level datasets through several approaches. First, efforts to link encounter-level data with comprehensive hospital characteristics would create powerful resources for research, capturing where children receive care and the service capabilities of those facilities. Second, expanding our models to incorporate additional variables, such as geographic indicators, or using spatial analysis techniques could further improve accuracy in identifying pediatric services. Third, the use of longitudinal, integrated multisource data would strengthen the reliability for tracking service changes over time.

### Limitations

This study has limitations include the high rates of missingness within the AHA data and differing definitions of service lines applied in each dataset. Therefore, we cannot determine whether the inconsistencies in data reporting relate to variable definitions or errors in data reporting. As discussed above, the availability of services does not reflect volumes of pediatric hospitalizations. Next, the use of the NPRP as the benchmark for a hospital-level administrative source is not without limitations. The NPRP is self-reported data, which may be influenced by social desirability and variability in the respondents. The NPRP questions on pediatric intensive care identified substantially more hospitals that reported this service than the AHA or the POS. This possibly represents hospitals that admit older adolescent patients to an adult intensive care unit after trauma, surgery, or medical conditions classically considered more adult-focused (eg, drug overdose). Next, although all datasets were from 2021, the timing of data collection within that year may not have aligned, potentially affecting service availability reporting. Lastly, our exploratory models are limited by the data we included. Future explorations using hierarchical models, spatial smoothing, or geographically weighted regressions may better approximate regional patterns.

## Conclusions

National hospital-level datasets varied substantially in variables used to define pediatric inpatient services and these variables did not consistently predict pediatric service availability. As large datasets are frequently used to inform health policy, a multi-dataset approach with careful attention to variable definitions may improve inference associated with pediatric hospital services. Accurate data that represent acute care hospital services for children are needed to inform policies that support access for children across the US. Such data could enable policymakers to identify gaps in care, allocate resources more effectively, and ensure that children, regardless of their geographic location, receive the timely and specialized care they need.

## Notes

Supplement 1. **eTable 1.** Data Processing Steps for Merging of the AHA, POS, and NPRP Datasets, Identification of Acute Care Hospitals **eTable 2.** Identification of Pediatric Services: Provision of Services Definition for Each Dataset **eAppendix 1.** Bias Assessment, Dataset Inclusion, Explanation of Variable Combinations **eAppendix 2.** Variables Included in Machine-Learning Models **eAppendix 3.** Exploratory Model Methods **eTable 3.** Discordant Statistics in the AHA and POS Using NPRP as the Gold Standard to Inform the Sequential Rule-Based Model **eTable 4.** Sequential Rule-Based Model **eTable 5.** Test Characteristics of Combinations of Variables for AHA and POS Compared With the NPRP **eTable 6.** Exploratory Model Test Characteristics of a Combined Dataset of AHA and POS Using the NPRP as the Gold Standard for Provision of Pediatric Services

Supplement 2.
Data Sharing Statement
