# Supplementary material for: Identifying Inpatient Pediatric Services Across National Datasets
Source: JAMA Netw Open. 2025 Jun 3;8(6):e2513527. doi: 10.1001/jamanetworkopen.2025.13527 (PMC12134956; doi:10.1001/jamanetworkopen.2025.13527)
Supplement: Supplement 1. — eTable 1. Data Processing Steps for Merging of the AHA, POS, and NPRP Datasets, Identification of Acute Care Hospitals eTable 2. Identification of Pediatric Services: Provision of Services Definition for Each Dataset eAppendix 1. Bias Assessment, Dataset Inclusion, Explanation of Variable Combinations eAppendix 2. Variables Included in Machine-Learning Models eAppendix 3. Exploratory Model Methods eTable 3. Discordant Statistics in the AHA and POS Using NPRP as the Gold Standard to Inform the Sequential Rule-Based Model eTable 4. Sequential Rule-Based Model eTable 5. Test Characteristics of Combinations of Variables for AHA and POS Compared With the NPRP eTable 6. Exploratory Model Test Characteristics of a Combined Dataset of AHA and POS Using the NPRP as the Gold Standard for Provision of Pediatric Services [file jamanetwopen-e2513527-s001.pdf]

# Supplemental Online Content

McDaniel CE, Ralston Daniel M, Freyleue SD, et al. Identifying inpatient pediatric services across national datasets. *JAMA Netw Open*. 2025;8(6):e2513527. doi:10.1001/jamanetworkopen.2025.13527

**eTable 1.** Data Processing Steps for Merging of the AHA, POS, and NPRP Datasets, Identification of Acute Care Hospitals

**eTable 2.** Identification of Pediatric Services: Provision of Services Definition for Each Dataset

**eAppendix 1.** Bias Assessment, Dataset Inclusion, Explanation of Variable Combinations

**eAppendix 2.** Variables Included in Machine-Learning Models

**eAppendix 3.** Exploratory Model Methods

**eTable 3.** Discordant Statistics in the AHA and POS Using NPRP as the Gold Standard to Inform the Sequential Rule-Based Model

**eTable 4.** Sequential Rule-Based Model

**eTable 5.** Test Characteristics of Combinations of Variables for AHA and POS Compared With the NPRP

**eTable 6.** Exploratory Model Test Characteristics of a Combined Dataset of AHA and POS Using the NPRP as the Gold Standard for Provision of Pediatric Services

This supplemental material has been provided by the authors to give readers additional information about their work.

© 2025 McDaniel CE et al. *JAMA Network Open*.

This supplemental material has been provided by the authors to give readers additional information about their work.

**eTable 1. Data Processing Steps for Merging of the AHA, POS, and NPRP Datasets, Identification of Acute Care Hospitals**

| Merging Process 2021 | Starting N (hospitals) | Finished N (Hospitals) |
|---|---|---|
| ***AHA + POS Merge*** | | |
| • ***Clean POS data preliminarily***<br>o POS limited to max quarter 4<br>o Limit to hospitals: filter(PRVDR_CTGRY_SBTYP_CD == 1)<br>o Limited to short term, CAH, Peds: filter(GNRL_FAC_TYPE_CD %in% c("1", “4”, "6", "11")) | 155,262 | After PRVDRCTGRY = 13,137<br><br>After PRVDR and then SHORT TERM = 10,623 |
| • **Merge partially cleaned POS data with raw AHA**<br>o Merge conducted by:<br>▪ 1. MCR_NUM & Zip matched<br>▪ 2. MCR_NUM & “city name” matched<br>▪ 3. MCR_NUM alone matched<br>▪ 4. Zip alone matched<br>▪ Duplicates filtered out by MCR_NUM and Zip codes<br>▪ 5. standardized hospital names by “upper case” and matched on name<br>▪ 6. standardized by matching street addresses standardized by “upper case”<br>▪ For hospitals that matched ONLY on MCR_NUM or ZIP, manually checked for verification | AHA: 6,201<br><br>POS: 10,623 | 5,507 |
| • **Cleaning of the merged AHA + POS**<br>o AHA Hospital Service Code: 10, 22, 50-59, 91<br>o Hospital in approved states: filter(!(STATE_CD %in% c("AS", "CN", "FM", "GU", “MH”, "MP", "PR", "VI")))<br>o Bed count > 1, as facilities with =<1 bed are stand alone ERs or OP surgery | 5,507 | 5,032 |

© 2025 McDaniel CE et al. *JAMA Network Open*.

| | | |
|---|---|---|
| ○ Removal of hospitals with CLOSED in the name<br>○ Provider Number service codes limited to PRVDR_NUM 1-879, 1300-1399, 3300-3399, 4000-4499<br>○ Match against GHL by CMS CCN<br>• **Manual removal of duplicates** à comparing cleaned AHA unmerged and cleaned POS unmerged with cleaned POS+AHA merged, checking for the below with manual google verification, with preference for keeping duplicates in the clean merged POS + AHA (fuzzy matching)<br>○ Duplicate CCNs<br>○ Standardizing by name & address within the same state | | |
| • **Cleaning of unmerged AHA and POS**<br>○ Unmerged AHA:<br>▪ Hospital Service Code: 10, 22, 50-59, 91<br>▪ Hospital in approved states: filter(!(STATE_CD %in% c("AS", "CN", "FM", "GU", "MH", "MP", "PR", "VI")))<br>▪ Bed count > 1, as facilities with =<1 bed are stand alone ERs or OP surgery<br>▪ Closed not in the name<br>▪ Provider Number service codes limited to PRVDR_NUM 1-879, 1300-1399, 3300-3399, 4000-4499<br>▪ Match against GHL by CMS CCN<br>○ Unmerged POS:<br>▪ Hospital in approved states: filter(!(STATE_CD %in% c("AS", "CN", "FM", "GU", "MH", "MP", "PR", "VI")))<br>▪ Bed count > 1, as facilities with =<1 bed are stand alone ERs or OP surgery<br>▪ Closed not in the name<br>▪ Provider Number service codes limited to PRVDR_NUM 1-879, 1300-1399, 3300-3399, 4000-4499<br>▪ Match against GHL by CMS CCN | AHAUNMERGED:460<br><br>POSUNMERGED: 5,116 | AHAUNMERGED:0<br><br>POSUNMERGED: 2,075 |
| • **Finalization of combined dataset of acute care + psych hospitals**<br>○ (Merged AHA + POS) + (Unmerged, clean AHA) + (Unmerged, clean POS) | 7,107 | 7,107 |
| ***NPRP + AHA + POS Merge*** | | |
| • **Merging of NPRP + CLMS** (NPRP's master file of hospitals)<br>○ CLMS merged onto NPRP by Portal ID<br>○ All NPRP hospitals kept | Clms: 4,633<br>NPRP: 3,646 | Merged: 3646 |
| • **Cleaning of NPRP + CLMS** | 3,646 | 3,400 |

© 2025 McDaniel CE et al. *JAMA Network Open*.

| | | |
|---|---|---|
| o Removal of non-approved states: nprpclms21 <- nprpclms21 %>% filter(nprpclms21$state != 'Puerto Rico')<br>o Removal of non-acute care hospitals: "FreeEDHosp" or "SATHosp" | | |
| • **Merge NPRP+CLMS with AHA+POS, uncleaned AHA unmerged, and uncleaned POS unmerged**<br>o Removal of psych hospitals (22 in AHA and 4 in POS and/or prvdr_num 4000-4499)<br>o 1. Standardized names between NPRP, CLMS, AHA, and POS (no punctuation, no spaces, or filler words)<br>o 2. Split address into 3 sections (4 addresses with 3 sections): street number, street name, street type (e.g., "rd")<br>o 3. "NPRP_CITY" = POS "CITY_NAME","NPRP_ADDRESS" = POS "ST_ADR","NPRP_NAME" = "AHA_NAME"<br>o 4. Repeat but with CLMS address<br>o 5. NPRP zip = AHA zip AND NPRP name = AHA name<br>o 6. NPRP name = AHA name but only with unique names<br>o 7. Matching by unique zipcodes alone<br>o 8. 3 parts of NPRP address matching 3 parts of AHA address, keeping only unique 1:1 matches<br>o 9. POS = CLMS in 3 parts of address<br>o 10. NPRP standardized name = AHA standardized name<br>o 11. NPRP name + number = AHA name + number, repeated with NPRP to POS and CLMS to AHA and POS<br>o 12. CLMS standardized name = AHA standardized name, POS standardized name, and NPRP to POS standardized<br>o 13. Unmerged NPRP/CLMS and the unmerged AHA/POS merged copied to proceed with fuzzy matches:<br>▪ Similarity threshold to 0.8 (due to probabilistic matching)<br>▪ jaro winkler algorithm<br>▪ Standardized names and address parts (all combinations)<br>▪ Fuzzy match combinations manually checked for verification<br>o Manual checking of merged NPRP hospitals that matched only on a single variable<br>o Manual checking of unmatched NPRP hospitals by state with AHA + POS, AHA unmerged, and POS unmerged (excluding fuzzy matches)<br>▪ Verification by hospital name, zip, address and confirmation through google searching<br>o Combined all confirmed files into the single merged file | NPRPCLMS: 3,400<br><br>AHAPOS: 5,032<br><br>AHAUNMERGED:460<br>POSUNMERGED: 5,116 | 3,086 AHAPOS merged to NPRPCLMS<br><br>Unmerged POS to NPRPCLMS after first merge 120<br><br>Unmerged AHA to NPRPCLMS after first and second merges, 3<br><br>184 observations in the nprpclms are left over and unmerged |

© 2025 McDaniel CE et al. *JAMA Network Open*.

| | | |
|---|---|---|
| • **Merging of final datasets**<br>o Merged NPRP + AHA + POS<br>o Unmerged NPRP-->merged onto unmerged POS<br>o Remaining unmerged NPRP-->merged onto unmerged AHA<br>o Remaining unmerged NPRP manually checked for finding in POS<br>▪ Fuzzy matching NPRP + POS by zipcode then manually checking<br>▪ Fuzzy matching NPRP + AHA by zipcode then manually checking<br>▪ Then reviewed manual checks | There were 92 manual matches | 3,114 was the size of the final merge after duplicates are removed |

© 2025 McDaniel CE et al. *JAMA Network Open*.

**eTable 2. Identification of Pediatric Services: Provision of Services Definition for Each Dataset**

| | | | NPRP | AHA | POS |
|---|---|---|---|---|---|
| Pediatric Services | Newborn care | | Yes to 18a, InptPedCap_Nursery_YN | OBBD≥1 OR OBHOS=1 OR OBLEV ≥1 | OB_SRVC_CD= 1, 2, or 3 |
| | SCN | Intermediate care | Yes to 18b, InptPedCap_NICU_YN | NINTBD≥1 or NINTHOS=1 | NEONTL_NRSRY_SRVC_CD = 1, 2, or 3 |
| | | NICU | | NICBD≥1 or NICHOS=1 | NEONTL_ICU_SRVC_CD = 1, 2, or 3 |
| | Wards | Peds only | Yes to 18e, InptPedCap_PedWard_YN | PEDBD≥1 or PEDHOS=1 | PED_SRVC_CD = 1, 2, or 3 |
| | | Unique Ped Only | Yes to 18e, InptPedCap_PedWard_YN And NO to to ChildAdmitAdultWard | | |
| | | Adult/Peds | Yes to 21 ChildAdmitAdultWard<br><br>Conditioned on (Yes to 18e,InptPedCap_PedWard_YN) | | |
| | | Unique Adult/Peds Only | Yes to ChildAdmitAdultWard AND No to 18e, InptPedCap_PedWard_YN | | |
| | PICU | Peds only | Yes to 18c, InptPedCap_PICU_YN | PEDICBD≥1 or PEDICHOS=1 | PED_ICU_SRVC_CD = 1, 2, or 3 |
| | | Unique Peds Only | Yes to 18c, InptPedCap_PICU_YN AND No to 19 ChildAdmitAdultICU | | |
| | | Adult/Peds | Yes to 19 ChildAdmitAdultICU<br><br>Conditioned on (Yes to 18c, InptPedCap_PICU_YN) | | |
| | | Unique Adult Peds Only | Yes to 19 ChildAdmitAdultICU AND No to 18c, InptPedCap_PICU_YN | | |
| | Psych | | n/a | PSYCABD≥1 or PSYCAHOS=1 | CHLD_ADLSCNT_PSYCH_SRVC_CD = 1, 2, or 3 |
| Pediatric serving | Any services (OR Logic) | | Yes to 18a InptPedCap_Nursery_YN OR 18b InptPedCap_NICU_YN OR 18c, InptPedCap_PICU_YN OR 18 e InptPedCap_PedWard_YN OR , or 19 ChildAdmitAdultICU or 21 ChildAdmitAdultWard | Yes to OBBD≥1, OBHOS=1, OBLEV ≥1, NINTBD≥1, NINTHOS=1, NICBD≥1, NICHOS=1, PEDBD≥1, PEDHOS=1, PEDICBD≥1, PEDICHOS=1, PSYCABD≥1, or PSYCAHOS=1 | Yes to [OB_SRVC_CD= 1, 2, or 3], [NEONTL_NRSRY_SRVC_CD = 1, 2, or 3], [NEONTL_ICU_SRVC_CD = 1, 2, or 3], [PED_SRVC_CD = 1, 2, or 3], [PED_ICU_SRVC_CD = 1, 2, or 3],OR [CHLD_ADLSCENT_PSYCH_SRVC_CD = 1, 2, or 3] |

© 2025 McDaniel CE et al. *JAMA Network Open*.

**eAppendix 1. Bias Assessment, Dataset Inclusion, Explanation of Variable Combinations**

***Bias Assessment and Dataset Inclusion***

We identified several potential sources of bias in our cross-sectional comparison. Selection bias was addressed by including all hospitals present across all three datasets rather than sampling, though this approach necessarily excluded hospitals not captured in all three sources. We mitigated information bias by standardizing variable definitions across datasets and conducting sensitivity analyses with alternative service line definitions. The voluntary nature of NPRP participation (compared to the more comprehensive but administratively-focused AHA and POS datasets) represents a potential source of reporting bias, as hospitals with more robust pediatric services may be more likely to participate in the NPRP. We partially addressed this by examining the characteristics of hospitals present in all datasets but missing data in specific ones.

Non-participation in our analysis occurred at several stages: (1) hospitals present in some but not all three datasets, (2) hospitals with complete missing data for service lines in any dataset, and (3) hospitals with specific missing variables needed for model development. The first group was excluded by design to enable direct comparison across datasets. The second group was necessary to exclude due to our complete case analysis approach for primary comparisons. The third group was only excluded from the exploratory modeling portion of the analysis.

***Missing Data and Variable Combinations***

Hospitals with missing service line data were excluded. The exception to this was the definition for newborn care in the AHA due to skip logic in the survey. Across all service lines, the AHA was missing service-line data from 796 hospitals (25.6%) and the POS was missing data from 26 hospitals (0.8%), all of which were excluded from this analysis. Given the

© 2025 McDaniel CE et al. *JAMA Network Open*.

structural rather than random missingness of the AHA data, we chose not to apply imputation techniques. While the POS and NPRP datasets had lower rates of missingness that might have technically allowed for imputation, we maintained consistent complete case analysis across all datasets to ensure methodological coherence in our comparative framework.

We evaluated all single variables (e.g., “AHA OB beds ≥ 1”) and two or more variables in combination with “ANDs” or “ORs” between variables (e.g., “AHA OB services AND OB beds ≥ 1 AND OB level ≥ 1”). In the AHA variables, newborn has 3 variables that when in combination results in 2520 possible combinations and NICU, which has 4 variables, results in 16,383 combinations. As such, we limited the analysis to logical combinations of ANDs and ORs of the variables, as decided by the research team. However, as a sensitivity analysis, we examined all potential combinations of AHA variables.

For newborn care, the NPRP has a single variable, “on-site provision of newborn care.” 18 hospitals were excluded due to missing NPRP data. The AHA has 3 possible variables, “OB services,” “OB beds ≥ 1,” and “OB level ≥ 1.” The POS has 1 variable, “OB services.” For this service line, we evaluated eleven combinations of variables from the AHA and the single combination of variables from the POS.

For neonatal intensive care, the NPRP again has a single variable, “on-site provision of neonatal intensive care.” Twenty-seven hospitals were excluded due to missing NPRP data. The AHA has 4 possible variables, “NICU services,” “NICU beds ≥0,” “neonatal intermediate care services,” and “neonatal intermediate care beds ≥0.” The POS has 2 possible variables, “Neonatal intensive care services,” and “neonatal intermediate care services.” We evaluated 26 combinations of AHA variables and 4 combinations of POS variables.

© 2025 McDaniel CE et al. *JAMA Network Open*.

For general pediatric inpatient services, we compared the pediatric-only and comprehensive definitions from the NPRP to the AHA and POS. Four hospitals were excluded for missing NPRP data. The pediatric-only and comprehensive definitions were compared to the 2 AHA variables independently and in combination as "AND" and "OR" ("general pediatric services" and "general pediatric care beds ≥1"), and to the single POS variable ("provision of pediatric services"). We evaluated 16 AHA variable combinations and 4 POS variable combinations.

Similarly, for pediatric intensive care services, we compared the pediatric-only and comprehensive NPRP definitions, excluding 12 hospitals for missing NPRP data. The AHA had 2 variables ("PICU services" and "PICU beds ≥1") and the POS had a single variable ("provision of pediatric intensive care"). We evaluated 16 AHA variable combinations and 4 POS variable combinations.

© 2025 McDaniel CE et al. *JAMA Network Open*.

**eAppendix 2. Variables Included in Machine-Learning Models**

```
data[['InptPedCap_Nursery_YN','InptPedCap_NICU_YN','InptPedCap_PICU_YN','ChildAdmitAdultIC
U','InptPedCap_PedWard_YN','ChildAdmitAdultWard','OB_SRVC_CD',
'NEONTL_NRSRY_SRVC_CD', 'NEONTL_ICU_SRVC_CD', 'PED_SRVC_CD',
'PED_ICU_SRVC_CD', 'CHLD_ADLSCNT_PSYCH_SRVC_CD', 'obbd', 'obhos', 'nintbd', 'ninthos',
'nicbd', 'nichos', 'pedbd', 'pedhos', 'pedicbd',  'pedichos', 'psycahos',
'PRVDR_CTGRY_SBTYP_CD', 'CMPLNC_STUS_CD', 'SKLTN_REC_SW', 'SSA_STATE_CD',
'GNRL_CNTL_TYPE_CD', 'GNRL_FAC_TYPE_CD', 'CRTFD_BED_CNT','CL_SRVC_CD',
'PHYSN_CNT','genhos','radmchi','pemerhos','psyphchos','alcpdhos', 'US Region', 'County']].
```

© 2025 McDaniel CE et al. *JAMA Network Open*.

**eAppendix 3. Exploratory Model Methods**

For the logistic regression model, we employed a non-hierarchical structure for the data without geographic predictors. In preliminary analyses, variance explained by geographic clustering was minimal compared to hospital-level characteristics. Furthermore, sensitivity analyses using clustered standard errors led to no difference in our primary findings.

For the machine learning models, we chose tree-based models for several reasons: (1) superior performance on structured tabular structured data, (2) interpretability requiring less hyperparameter tuning improving computational efficiency, and (3) proven reliability within healthcare analyses. To develop and validate the models, we divided the merged AHA-POS dataset into training (80%) and test (20%) sets, with the test set reserved exclusively for final model evaluation. Within the training set, we performed hyperparameter tuning using grid search with 10-fold cross-validation to optimize model parameters while minimizing overfitting risk.

To ensure model specificity, we selected relevant predictor variables. For NICU predictions, we included "NICU services" and "NICU beds ≥0" from AHA and from POS, we included "Neonatal intensive care services." For PICU predictions, we limited the NPRP variable to "On-site provision of pediatric intensive care within a designated pediatric intensive care unit" to maintain close alignment with corresponding AHA and POS variables. Importantly, we did not exclude hospitals with missing data to better reflect real-world use cases. Additionally, we enhanced our models by incorporating spatial components (US region, State, and County) to account for geographic variation. Throughout model development, we monitored performance metrics across both training and test sets to ensure consistent performance.

Lastly, we created a rule-based sequential reasoning process to predict services. Incorporating patterns from the machine learning algorithms with subject matter expertise, we

© 2025 McDaniel CE et al. *JAMA Network Open*.

developed a set of logical conditions. First, we assessed when the AHA and the POS were discordant across each service line, e.g., when the AHA data show a particular hospital has newborn services, but the POS data do not. Using these discordant pairs, we calculated true positives, true negatives, false positives, and false negatives relative to the NPRP for the AHA and POS (**Supplement 6**). This informed calculations of accuracy for the sequential rule-based model with and without missing data for each dataset.

We then established service line specific rules to determine when either the AHA or POS should be used to predict the provision of services (as recorded in the NPRP), in cases of discordance (**Supplement 7**). We intentionally established rules that would emphasize prediction sensitivity over specificity. For example, if a hospital provided neonatal intensive care according either the AHA or POS, we assumed that hospital indeed provided the service. This resulted in four outcomes for each service line: (1) AHA + POS agree there is existence of a service line, (2) AHA + POS agree there is not existence of a service line, (3) AHA + POS are discordant, and (4) no data. In the third scenario, the established rules were applied sequentially.

Using the 20% test-set from in the machine learning models, we evaluated the performance of the logistic regression model, both tree-based models, and the rule-based sequential reasoning against the NPRP, establishing test statistics for each dataset combination used for prediction.

© 2025 McDaniel CE et al. *JAMA Network Open*.

**eTable 3:** Discordant Statistics in the AHA and POS Using NPRP as the Gold Standard to Inform the Sequential Rule-Based Model

| **Service Line** | **AHA** indicates a hospital has indicated service line | **POS** indicates a hospital has indicated service line | **AHA** when AHA is missing (assumes missing = no) | **POS** when AHA is missing (assumes missing = no) | **Sequential Rule-Based Model** with missing data | **Sequential Rule-Based Model** excluding missing data |
|---|---|---|---|---|---|---|
| Newborn | • Accuracy: 85.11%<br>• Misclassification Rate: 14.89%<br>• Confusion Matrix:<br>○ TP: 40<br>○ TN: 0<br>○ FP: 7<br>○ FN: 0 | • Accuracy: 89.19%<br>• Misclassification Rate: 10.81%<br>• Confusion Matrix:<br>○ TP: 16<br>○ TN: 0<br>○ FP: 132<br>○ FN: 0 | • Accuracy: 51.51%<br>• Confusion Matrix:<br>○ TP: 0<br>○ TN: 410<br>○ FP: 0<br>○ FN: 386 | • Accuracy: 85.43%<br>• Confusion Matrix:<br>○ TP: 357<br>○ TN: 323<br>○ FP: 87<br>○ FN: 29 | • Accuracy: 84.36%<br>• Confusion Matrix:<br>○ TP: 1460<br>○ TN: 1167<br>○ FP: 75<br>○ FN: 412 | • Accuracy: 93.53%<br>• Confusion Matrix:<br>○ TP: 1817<br>○ TN: 1072<br>○ FP: 162<br>○ FN: 38 |
| Neonatal | • Accuracy: 75.38%<br>• Misclassification Rate: 24.62%<br>• Confusion Matrix:<br>○ TP: 49<br>○ TN: 0<br>○ FP: 16<br>○ FN: 0 | • Accuracy: 63.23%<br>• Misclassification Rate: 36.77%<br>• Confusion Matrix:<br>○ TP: 57<br>○ TN: 0<br>○ FP: 98<br>○ FN: 0 | • Accuracy: 51.51%<br>• Confusion Matrix:<br>○ TP: 0<br>○ TN: 410<br>○ FP: 0<br>○ FN: 386 | • Accuracy: 75.25%<br>• Confusion Matrix:<br>○ TP: 105<br>○ TN: 395<br>○ FP: 15<br>○ FN: 281 | • Accuracy: 79.09%<br>• Confusion Matrix:<br>○ TP: 1427<br>○ TN: 2283<br>○ FP: 206<br>○ FN: 445 | • Accuracy: 79.32%<br>• Confusion Matrix:<br>○ TP: 1168<br>○ TN: 670<br>○ FP: 161<br>○ FN: 318 |
| General Pediatrics | • Accuracy: 67.89%<br>• Misclassification Rate: 32.11%<br>• Confusion Matrix:<br>○ TP: 74<br>○ TN: 0<br>○ FP: 35<br>○ FN: 0 | • Accuracy: 41.06%<br>• Misclassification Rate: 58.94%<br>• Confusion Matrix:<br>○ TP: 432<br>○ TN: 0<br>○ FP: 301<br>○ FN: 0 | • Accuracy: 33.54%<br>• Confusion Matrix:<br>○ TP: 0<br>○ TN: 267<br>○ FP: 0<br>○ FN: 529 | • Accuracy: 64.95%<br>• Confusion Matrix:<br>○ TP: 378<br>○ TN: 139<br>○ FP: 128<br>○ FN: 151 | • Accuracy: 69.65%<br>• Confusion Matrix:<br>○ TP: 1718<br>○ TN: 451<br>○ FP: 573<br>○ FN: 372 | • Accuracy: 71.95%<br>• Confusion Matrix:<br>○ TP: 1407<br>○ TN: 312<br>○ FP: 450<br>○ FN: 220 |
| Pediatric Intensive Care | • Accuracy: 88.30%<br>• Misclassification Rate: 11.70%<br>• Confusion Matrix:<br>○ TP: 151<br>○ FN: 0<br>○ FP: 20<br>○ FN: 0 | • Accuracy: 19.15%<br>• Misclassification Rate: 80.85%<br>• Confusion Matrix:<br>○ TP: 76<br>○ TN: 0<br>○ FP: 18<br>○ FN: 0 | • Accuracy: 78.14%<br>• Confusion Matrix:<br>○ TP: 0<br>○ TN: 622<br>○ FP: 0<br>○ FN: 174 | • Accuracy: 62.81%<br>• Confusion Matrix:<br>○ TP: 49<br>○ TN: 599<br>○ FP: 23<br>○ FN: 125 | • Accuracy: 80.10%<br>• Confusion Matrix:<br>○ TP: 233<br>○ TN: 2283<br>○ FP: 26<br>○ FN: 572 | • Accuracy: 81.94%<br>• Confusion Matrix:<br>○ TP: 282<br>○ TN: 2249<br>○ FP: 49<br>○ FN: 509 |

© 2025 McDaniel CE et al. *JAMA Network Open*.

**eTable 4.** Sequential Rule-Based Model

| Service-line | (+) Service definition | (-) Service definition |
|---|---|---|
| Newborn care | AHA = 1, OR<br>AHA = missing & POS = 1 | AHA = 0, OR<br>AHA = missing & POS = 0 |
| Neonatal intensive care | AHA =1, OR<br>POS =1 | AHA and POS = 0<br>AHA = missing & POS =0<br>POS = missing & AHA = 0 |
| General pediatric care | AHA =1, OR<br>POS =1 | AHA and POS = 0<br>AHA = missing & POS =0<br>POS = missing & AHA = 0 |
| Pediatric intensive care | AHA = 1, OR<br>AHA = missing & POS = 1 | AHA = 0, OR<br>AHA = missing & POS = 0 |

Unknown: AHA & POS = Missing

© 2025 McDaniel CE et al. *JAMA Network Open*.

## eTable 5. Test Characteristics of Combinations of Variables for AHA and POS Compared With the NPRP

| NPRP Variable | AHA or POS Variable | F1 | F1_CI | Sensitivity | Sensitivity_CI | Specificity | Specificity_CI | PPV | PPV_CI | NPV | NPV_CI | LR+ | LR+_CI | LR- | LR-_CI |
|---|---|---|---|---|---|---|---|---|---|---|---|---|---|---|---|
| Newborn Care: AHA to NPRP | | | | | | | | | | | | | | | |
| nprp_nursery | aha_nursery_obhos | 0.97 | (0.96, 0.97) | 0.98 | (0.98, 0.99) | 0.91 | (0.89, 0.93) | 0.95 | (0.94, 0.96) | 0.97 | (0.95, 0.98) | 10.89 | (8.77, 13.51) | 0.02 | (0.01, 0.03) |
| nprp_nursery | aha_nursery_oblev | 0.97 | (0.96, 0.97) | 0.98 | (0.98, 0.99) | 0.91 | (0.89, 0.93) | 0.95 | (0.94, 0.96) | 0.97 | (0.95, 0.98) | 10.89 | (8.77, 13.51) | 0.02 | (0.01, 0.03) |
| nprp_nursery | aha_nursery_obbd OR.aha_nursery_obhos | 0.97 | (0.96, 0.97) | 0.98 | (0.98, 0.99) | 0.91 | (0.89, 0.93) | 0.95 | (0.94, 0.96) | 0.97 | (0.95, 0.98) | 10.89 | (8.77, 13.51) | 0.02 | (0.01, 0.03) |
| nprp_nursery | aha_nursery_obbd OR.aha_nursery_oblev | 0.97 | (0.96, 0.97) | 0.98 | (0.98, 0.99) | 0.91 | (0.89, 0.93) | 0.95 | (0.94, 0.96) | 0.97 | (0.95, 0.98) | 10.89 | (8.77, 13.51) | 0.02 | (0.01, 0.03) |
| nprp_nursery | aha_nursery_obhos OR.aha_nursery_oblev | 0.97 | (0.96, 0.97) | 0.98 | (0.98, 0.99) | 0.91 | (0.89, 0.93) | 0.95 | (0.94, 0.96) | 0.97 | (0.95, 0.98) | 10.89 | (8.77, 13.51) | 0.02 | (0.01, 0.03) |
| nprp_nursery | aha_nursery_obhos AND.aha_nursery_oblev | 0.97 | (0.96, 0.97) | 0.98 | (0.98, 0.99) | 0.91 | (0.89, 0.93) | 0.95 | (0.94, 0.96) | 0.97 | (0.95, 0.98) | 10.89 | (8.77, 13.51) | 0.02 | (0.01, 0.03) |
| nprp_nursery | aha_nursery_obbd OR.aha_nursery_obho sOR.aha_nursery_oblev | 0.97 | (0.96, 0.97) | 0.98 | (0.98, 0.99) | 0.91 | (0.89, 0.93) | 0.95 | (0.94, 0.96) | 0.97 | (0.95, 0.98) | 10.89 | (8.77, 13.51) | 0.02 | (0.01, 0.03) |
| nprp_nursery | aha_nursery_obbd | 0.96 | (0.95, 0.97) | 0.97 | (0.96, 0.98) | 0.91 | (0.89, 0.93) | 0.95 | (0.94, 0.96) | 0.95 | (0.93, 0.96) | 11.05 | (8.87, 13.75) | 0.03 | (0.02, 0.04) |
| nprp_nursery | aha_nursery_obbd AND.aha_nursery_obhos | 0.96 | (0.95, 0.97) | 0.97 | (0.96, 0.98) | 0.91 | (0.89, 0.93) | 0.95 | (0.94, 0.96) | 0.95 | (0.93, 0.96) | 11.05 | (8.87, 13.75) | 0.03 | (0.02, 0.04) |
| nprp_nursery | aha_nursery_obbd AND.aha_nursery_oblev | 0.96 | (0.95, 0.97) | 0.97 | (0.96, 0.98) | 0.91 | (0.89, 0.93) | 0.95 | (0.94, 0.96) | 0.95 | (0.93, 0.96) | 11.05 | (8.87, 13.75) | 0.03 | (0.02, 0.04) |
| nprp_nursery | aha_nursery_obbd AND.aha_nursery_obhos AND.aha_nursery_oblev | 0.96 | (0.95, 0.97) | 0.97 | (0.96, 0.98) | 0.91 | (0.89, 0.93) | 0.95 | (0.94, 0.96) | 0.95 | (0.93, 0.96) | 11.05 | (8.87, 13.75) | 0.03 | (0.02, 0.04) |
| Nursery: POS to NPRP | | | | | | | | | | | | | | | |
| nprp_nursery | pos_nursery | 0.92 | (0.91, 0.93) | 0.97 | (0.96, 0.98) | 0.76 | (0.73, 0.79) | 0.88 | (0.86, 0.89) | 0.93 | (0.91, 0.95) | 4.02 | (3.56, 4.53) | 0.04 | (0.03, 0.06) |
| Neonatal Intensive Care: AHA to NPRP | | | | | | | | | | | | | | | |
| nprp_nicu | aha_nicu_nicbd OR.aha_nicu_nintbd | 0.86 | (0.85, 0.88) | 0.92 | (0.90, 0.94) | 0.91 | (0.89, 0.92) | 0.82 | (0.79, 0.84) | 0.96 | (0.95, 0.97) | 9.79 | (8.39, 11.43) | 0.09 | (0.07, 0.12) |
| nprp_nicu | aha_nicu_nichos OR.aha_nicu_nintbd | 0.86 | (0.85, 0.88) | 0.93 | (0.91, 0.95) | 0.90 | (0.88, 0.91) | 0.81 | (0.78, 0.83) | 0.96 | (0.96, 0.97) | 9.11 | (7.86, 10.56) | 0.08 | (0.06, 0.10) |
| nprp_nicu | aha_nicu_nicbd OR.aha_nicu_nichos OR.aha_nicu_nintbd | 0.86 | (0.85, 0.88) | 0.93 | (0.91, 0.95) | 0.90 | (0.88, 0.91) | 0.81 | (0.78, 0.83) | 0.96 | (0.96, 0.97) | 9.11 | (7.86, 10.56) | 0.08 | (0.06, 0.10) |
| nprp_nicu | aha_nicu_nichos | 0.86 | (0.85, 0.87) | 0.82 | (0.80, 0.85) | 0.96 | (0.95, 0.97) | 0.90 | (0.88, 0.92) | 0.92 | (0.91, 0.94) | 19.85 | (15.64, 25.20) | 0.18 | (0.16, 0.22) |
| nprp_nicu | aha_nicu_nicbd OR.aha_nicu_nichos | 0.86 | (0.85, 0.87) | 0.82 | (0.80, 0.85) | 0.96 | (0.95, 0.97) | 0.90 | (0.88, 0.92) | 0.92 | (0.91, 0.94) | 19.85 | (15.64, 25.20) | 0.18 | (0.16, 0.22) |
| nprp_nicu | aha_nicu_nicbd | 0.86 | (0.84, 0.87) | 0.81 | (0.78, 0.83) | 0.97 | (0.96, 0.98) | 0.92 | (0.90, 0.94) | 0.92 | (0.90, 0.93) | 24.65 | (18.82, 32.28) | 0.20 | (0.17, 0.23) |
| nprp_nicu | aha_nicu_nicbd AND.aha_nicu_nichos | 0.86 | (0.84, 0.87) | 0.81 | (0.78, 0.83) | 0.97 | (0.96, 0.98) | 0.92 | (0.90, 0.94) | 0.92 | (0.90, 0.93) | 24.65 | (18.82, 32.28) | 0.20 | (0.17, 0.23) |

© 2025 McDaniel CE et al. *JAMA Network Open*.

| | | | | | | | | | | | | | | | |
|---|---|---|---|---|---|---|---|---|---|---|---|---|---|---|---|
| nprp_nicu | aha_nicu_nicbd OR.aha_nicu_ninthos | 0.85 | (0.84, 0.87) | 0.93 | (0.91, 0.95) | 0.89 | (0.87, 0.90) | 0.79 | (0.76, 0.82) | 0.97 | (0.96, 0.98) | 8.14 | (7.09, 9.34) | 0.08 | (0.06, 0.10) |
| nprp_nicu | aha_nicu_nicbd OR.aha_nicu_nintbdOR.aha_nicu_ninthos | 0.85 | (0.84, 0.87) | 0.93 | (0.91, 0.95) | 0.89 | (0.87, 0.90) | 0.79 | (0.76, 0.82) | 0.97 | (0.96, 0.98) | 8.14 | (7.09, 9.34) | 0.08 | (0.06, 0.10) |
| nprp_nicu | aha_nicu_nichosOR.aha_nicu_ninthos | 0.85 | (0.84, 0.87) | 0.94 | (0.92, 0.95) | 0.88 | (0.87, 0.90) | 0.78 | (0.76, 0.81) | 0.97 | (0.96, 0.98) | 7.95 | (6.94, 9.11) | 0.07 | (0.06, 0.10) |
| nprp_nicu | aha_nicu_nicbd OR.aha_nicu_nichos OR.aha_nicu_ninthos | 0.85 | (0.84, 0.87) | 0.94 | (0.92, 0.95) | 0.88 | (0.87, 0.90) | 0.78 | (0.76, 0.81) | 0.97 | (0.96, 0.98) | 7.95 | (6.94, 9.11) | 0.07 | (0.06, 0.10) |
| nprp_nicu | aha_nicu_nichos OR.aha_nicu_nintbd OR.aha_nicu_ninthos | 0.85 | (0.84, 0.87) | 0.94 | (0.92, 0.95) | 0.88 | (0.87, 0.90) | 0.78 | (0.76, 0.81) | 0.97 | (0.96, 0.98) | 7.95 | (6.94, 9.11) | 0.07 | (0.06, 0.10) |
| nprp_nicu | aha_nicu_nicbd OR.aha_nicu_nichos OR.aha_nicu_nintbdOR.aha_nicu_ninthos | 0.85 | (0.84, 0.87) | 0.94 | (0.92, 0.95) | 0.88 | (0.87, 0.90) | 0.78 | (0.76, 0.81) | 0.97 | (0.96, 0.98) | 7.95 | (6.94, 9.11) | 0.07 | (0.06, 0.10) |
| nprp_nicu | aha_nicu_ninthos | 0.50 | (0.48, 0.52) | 0.40 | (0.37, 0.44) | 0.91 | (0.89, 0.92) | 0.67 | (0.62, 0.71) | 0.77 | (0.75, 0.79) | 4.37 | (3.66, 5.23) | 0.66 | (0.62, 0.70) |
| nprp_nicu | aha_nicu_nintbd OR.aha_nicu_ninthos | 0.50 | (0.48, 0.52) | 0.40 | (0.37, 0.44) | 0.91 | (0.89, 0.92) | 0.67 | (0.62, 0.71) | 0.77 | (0.75, 0.79) | 4.37 | (3.66, 5.23) | 0.66 | (0.62, 0.70) |
| nprp_nicu | aha_nicu_nichos AND.aha_nicu_ninthos | 0.44 | (0.42, 0.46) | 0.29 | (0.26, 0.32) | 0.98 | (0.98, 0.99) | 0.89 | (0.85, 0.93) | 0.75 | (0.73, 0.77) | 18.46 | (12.31, 27.68) | 0.72 | (0.69, 0.76) |
| nprp_nicu | aha_nicu_nintbd | 0.43 | (0.41, 0.45) | 0.31 | (0.28, 0.35) | 0.93 | (0.92, 0.94) | 0.67 | (0.62, 0.72) | 0.75 | (0.73, 0.77) | 4.51 | (3.66, 5.56) | 0.74 | (0.70, 0.78) |
| nprp_nicu | aha_nicu_nintbd AND.aha_nicu_ninthos | 0.43 | (0.41, 0.45) | 0.31 | (0.28, 0.35) | 0.93 | (0.92, 0.94) | 0.67 | (0.62, 0.72) | 0.75 | (0.73, 0.77) | 4.51 | (3.66, 5.56) | 0.74 | (0.70, 0.78) |
| nprp_nicu | aha_nicu_nicbd AND.aha_nicu_ninthos | 0.43 | (0.41, 0.45) | 0.28 | (0.24, 0.31) | 0.99 | (0.99, 0.99) | 0.93 | (0.89, 0.96) | 0.75 | (0.73, 0.77) | 27.48 | (16.64, 45.37) | 0.73 | (0.70, 0.76) |
| nprp_nicu | aha_nicu_nicbd AND.aha_nicu_nichos AND.aha_nicu_ninthos | 0.43 | (0.41, 0.45) | 0.28 | (0.24, 0.31) | 0.99 | (0.99, 0.99) | 0.93 | (0.89, 0.96) | 0.75 | (0.73, 0.77) | 27.48 | (16.64, 45.37) | 0.73 | (0.70, 0.76) |
| nprp_nicu | aha_nicu_nichos AND.aha_nicu_nintbd | 0.34 | (0.32, 0.36) | 0.21 | (0.18, 0.24) | 0.99 | (0.99, 1.00) | 0.91 | (0.87, 0.95) | 0.73 | (0.71, 0.75) | 22.31 | (13.22, 37.64) | 0.80 | (0.77, 0.83) |
| nprp_nicu | aha_nicu_nichos AND.aha_nicu_nintbd AND.aha_nicu_ninthos | 0.34 | (0.32, 0.36) | 0.21 | (0.18, 0.24) | 0.99 | (0.99, 1.00) | 0.91 | (0.87, 0.95) | 0.73 | (0.71, 0.75) | 22.31 | (13.22, 37.64) | 0.80 | (0.77, 0.83) |
| nprp_nicu | aha_nicu_nicbd AND.aha_nicu_nintbd | 0.33 | (0.31, 0.35) | 0.20 | (0.17, 0.23) | 0.99 | (0.99, 1.00) | 0.91 | (0.87, 0.96) | 0.73 | (0.71, 0.75) | 23.12 | (13.46, 39.71) | 0.80 | (0.77, 0.83) |
| nprp_nicu | aha_nicu_nicbd AND.aha_nicu_nichos AND.aha_nicu_nintbd | 0.33 | (0.31, 0.35) | 0.20 | (0.17, 0.23) | 0.99 | (0.99, 1.00) | 0.91 | (0.87, 0.96) | 0.73 | (0.71, 0.75) | 23.12 | (13.46, 39.71) | 0.80 | (0.77, 0.83) |
| nprp_nicu | aha_nicu_nicbd AND.aha_nicu_nintbd AND.aha_nicu_ninthos | 0.33 | (0.31, 0.35) | 0.20 | (0.17, 0.23) | 0.99 | (0.99, 1.00) | 0.91 | (0.87, 0.96) | 0.73 | (0.71, 0.75) | 23.12 | (13.46, 39.71) | 0.80 | (0.77, 0.83) |
| nprp_nicu | aha_nicu_nicbd AND.aha_nicu_nichos AND.aha_nicu_nintbd AND.aha_nicu_ninthos | 0.33 | (0.31, 0.35) | 0.20 | (0.17, 0.23) | 0.99 | (0.99, 1.00) | 0.91 | (0.87, 0.96) | 0.73 | (0.71, 0.75) | 23.12 | (13.46, 39.71) | 0.80 | (0.77, 0.83) |

© 2025 McDaniel CE et al. *JAMA Network Open*.

| | | | | | | | | | | | | | | | |
|---|---|---|---|---|---|---|---|---|---|---|---|---|---|---|---|
| Neonatal Intensive Care: POS to NPRP | | | | | | | | | | | | | | | |
| nprp_nicu | pos_nicu_icu | 0.75 | (0.74, 0.77) | 0.68 | (0.65, 0.71) | 0.94 | (0.93, 0.95) | 0.84 | (0.81, 0.87) | 0.87 | (0.85, 0.88) | 11.64 | (9.50, 14.27) | 0.34 | (0.30, 0.38) |
| nprp_nicu | pos_nicu_icu AND.pos_nicu_nursery | 0.74 | (0.72, 0.76) | 0.65 | (0.62, 0.69) | 0.95 | (0.94, 0.96) | 0.85 | (0.82, 0.88) | 0.86 | (0.84, 0.87) | 12.22 | (9.87, 15.13) | 0.37 | (0.33, 0.40) |
| nprp_nicu | pos_nicu_icu OR.pos_nicu_nursery | 0.68 | (0.66, 0.70) | 0.92 | (0.90, 0.94) | 0.64 | (0.62, 0.66) | 0.54 | (0.51, 0.57) | 0.95 | (0.93, 0.96) | 2.56 | (2.39, 2.74) | 0.12 | (0.10, 0.16) |
| nprp_nicu | pos_nicu_nursery | 0.67 | (0.65, 0.69) | 0.89 | (0.87, 0.92) | 0.65 | (0.62, 0.67) | 0.54 | (0.51, 0.56) | 0.93 | (0.91, 0.94) | 2.52 | (2.34, 2.70) | 0.17 | (0.13, 0.21) |
| General Pediatric Care: AHA to NPRP | | | | | | | | | | | | | | | |
| nprp_pediatrics_InptPedCap _PedWard_YN | aha_pediatrics_pedbd | 0.76 | (0.74, 0.78) | 0.74 | (0.71, 0.78) | 0.89 | (0.88, 0.91) | 0.78 | (0.75, 0.81) | 0.87 | (0.86, 0.89) | 6.96 | (5.97, 8.11) | 0.29 | (0.25, 0.33) |
| nprp_pediatrics_InptPedCap _PedWard_YN | aha_pediatrics_pedhos AND.aha_pediatrics_pedbd | 0.76 | (0.74, 0.78) | 0.74 | (0.71, 0.78) | 0.89 | (0.88, 0.91) | 0.78 | (0.75, 0.81) | 0.87 | (0.86, 0.89) | 6.96 | (5.97, 8.11) | 0.29 | (0.25, 0.33) |
| nprp_pediatrics_InptPedCap _PedWard_YN | aha_pediatrics_pedhos | 0.71 | (0.69, 0.73) | 0.84 | (0.81, 0.86) | 0.74 | (0.72, 0.77) | 0.62 | (0.59, 0.65) | 0.90 | (0.88, 0.92) | 3.26 | (2.97, 3.58) | 0.22 | (0.19, 0.26) |
| nprp_pediatrics_InptPedCap _PedWard_YN | aha_pediatrics_pedhosOR.aha_pediatrics_pedbd | 0.71 | (0.69, 0.73) | 0.84 | (0.81, 0.86) | 0.74 | (0.72, 0.77) | 0.62 | (0.59, 0.65) | 0.90 | (0.88, 0.92) | 3.26 | (2.97, 3.58) | 0.22 | (0.19, 0.26) |
| nprp_pediatrics_InptPedCap _PedWard_YN OR.nprp_pediatrics_ChildAdmit AdultWard | aha_pediatrics_pedhos | 0.69 | (0.67, 0.71) | 0.57 | (0.55, 0.60) | 0.82 | (0.79, 0.85) | 0.88 | (0.85, 0.90) | 0.47 | (0.44, 0.50) | 3.19 | (2.71, 3.77) | 0.52 | (0.49, 0.56) |
| nprp_pediatrics_InptPedCap _PedWard_YN OR.nprp_pediatrics_ChildAdmit AdultWar | aha_pediatrics_pedhos OR.aha_pediatrics_pedbd | 0.69 | (0.67, 0.71) | 0.57 | (0.55, 0.60) | 0.82 | (0.79, 0.85) | 0.88 | (0.85, 0.90) | 0.47 | (0.44, 0.50) | 3.19 | (2.71, 3.77) | 0.52 | (0.49, 0.56) |
| nprp_pediatrics_InptPedCap_ PedWard_YN OR.nprp_pediatrics_ChildAdmit AdultWard | aha_pediatrics_pedbd | 0.58 | (0.56, 0.60) | 0.43 | (0.40, 0.45) | 0.92 | (0.90, 0.94) | 0.92 | (0.90, 0.94) | 0.42 | (0.40, 0.45) | 5.27 | (4.08, 6.83) | 0.62 | (0.59, 0.65) |
| nprp_pediatrics_InptPedCap_PedWard_YNOR.nprp_pediatrics_ChildAdmit AdultWard | aha_pediatrics_pedhos AND.aha_pediatrics_pedbd | 0.58 | (0.56, 0.60) | 0.43 | (0.40, 0.45) | 0.92 | (0.90, 0.94) | 0.92 | (0.90, 0.94) | 0.42 | (0.40, 0.45) | 5.27 | (4.08, 6.83) | 0.62 | (0.59, 0.65) |
| nprp_pediatrics_ChildAdmit AdultWard | aha_pediatrics_pedhos | 0.47 | (0.44, 0.49) | 0.44 | (0.41, 0.47) | 0.54 | (0.51, 0.57) | 0.49 | (0.46, 0.52) | 0.50 | (0.47, 0.53) | 0.97 | (0.89, 1.07) | 1.02 | (0.95, 1.10) |
| nprp_pediatrics_ChildAdmit AdultWard | aha_pediatrics_pedhos OR.aha_pediatrics_pedbd | 0.47 | (0.44, 0.49) | 0.44 | (0.41, 0.47) | 0.54 | (0.51, 0.57) | 0.49 | (0.46, 0.52) | 0.50 | (0.47, 0.53) | 0.97 | (0.89, 1.07) | 1.02 | (0.95, 1.10) |
| nprp_pediatrics_InptPedCap_ PedWard_YN AND.nprp_pediatrics_ChildAdmit AdultWard | aha_pediatrics_pedbd | 0.36 | (0.34, 0.38) | 0.58 | (0.53, 0.64) | 0.72 | (0.70, 0.74) | 0.26 | (0.23, 0.29) | 0.91 | (0.90, 0.93) | 2.10 | (1.87, 2.37) | 0.58 | (0.51, 0.66) |

© 2025 McDaniel CE et al. *JAMA Network Open*.

| | | | | | | | | | | | | | | | |
|---|---|---|---|---|---|---|---|---|---|---|---|---|---|---|---|
| nprp_pediatrics_InptPedCap_ PedWard_YN AND.nprp_pediatrics_ChildAdmit AdultWard | aha_pediatrics_pedhos AND.aha_pediatrics_pedbd | 0.36 | (0.34, 0.38) | 0.58 | (0.53, 0.64) | 0.72 | (0.70, 0.74) | 0.26 | (0.23, 0.29) | 0.91 | (0.90, 0.93) | 2.10 | (1.87, 2.37) | 0.58 | (0.51, 0.66) |
| nprp_pediatrics_InptPedCap_ PedWard_YN AND.nprp_pediatrics_ChildAdmit AdultWard | aha_pediatrics_pedhos | 0.36 | (0.34, 0.38) | 0.74 | (0.69, 0.79) | 0.60 | (0.58, 0.62) | 0.23 | (0.21, 0.26) | 0.93 | (0.92, 0.95) | 1.84 | (1.69, 2.00) | 0.44 | (0.36, 0.53) |
| nprp_pediatrics_InptPedCap_ PedWard_YN AND.nprp_pediatrics_ ChildAdmitAdultWard | aha_pediatrics_pedhos OR.aha_pediatrics_pedbd | 0.36 | (0.34, 0.38) | 0.74 | (0.69, 0.79) | 0.60 | (0.58, 0.62) | 0.23 | (0.21, 0.26) | 0.93 | (0.92, 0.95) | 1.84 | (1.69, 2.00) | 0.44 | (0.36, 0.53) |
| nprp_pediatrics_ChildAdmit AdultWard | aha_pediatrics_pedbd | 0.32 | (0.30, 0.34) | 0.26 | (0.23, 0.29) | 0.62 | (0.59, 0.65) | 0.40 | (0.37, 0.44) | 0.46 | (0.44, 0.49) | 0.69 | (0.60, 0.78) | 1.19 | (1.12, 1.26) |
| nprp_pediatrics_ChildAdmit AdultWard | aha_pediatrics_pedhos AND.aha_pediatrics_pedbd | 0.32 | (0.30, 0.34) | 0.26 | (0.23, 0.29) | 0.62 | (0.59, 0.65) | 0.40 | (0.37, 0.44) | 0.46 | (0.44, 0.49) | 0.69 | (0.60, 0.78) | 1.19 | (1.12, 1.26) |
| General Pediatric Care: POS to NPRP | | | | | | | | | | | | | | | |
| nprp_pediatrics_InptPedCap_ PedWard_YN OR.nprp_pediatrics_ChildAdmit AdultWard | pos_pediatrics | 0.79 | (0.77, 0.80) | 0.81 | (0.79, 0.83) | 0.46 | (0.42, 0.50) | 0.77 | (0.75, 0.79) | 0.52 | (0.48, 0.56) | 1.50 | (1.39, 1.61) | 0.42 | (0.36, 0.47) |
| nprp_pediatrics_ChildAdmit AdultWard | pos_pediatrics | 0.61 | (0.59, 0.63) | 0.75 | (0.72, 0.77) | 0.30 | (0.27, 0.33) | 0.51 | (0.49, 0.54) | 0.55 | (0.51, 0.59) | 1.07 | (1.01, 1.13) | 0.84 | (0.73, 0.96) |
| nprp_pediatrics_InptPedCap_ PedWard_YN | pos_pediatrics | 0.59 | (0.57, 0.61) | 0.94 | (0.92, 0.96) | 0.38 | (0.36, 0.41) | 0.43 | (0.41, 0.46) | 0.93 | (0.91, 0.95) | 1.53 | (1.46, 1.60) | 0.16 | (0.12, 0.21) |
| nprp_pediatrics_InptPedCap_ PedWard_YN AND.nprp_pediatrics_ChildAdmit AdultWard | pos_pediatrics | 0.30 | (0.28, 0.32) | 0.91 | (0.88, 0.94) | 0.31 | (0.29, 0.33) | 0.18 | (0.16, 0.20) | 0.95 | (0.94, 0.97) | 1.31 | (1.25, 1.37) | 0.30 | (0.21, 0.43) |
| Pediatric Intensive Care: AHA to NPRP | | | | | | | | | | | | | | | |
| nprp_picu_InptPedCap_ PICU_YN | aha_picu_pedicbd | 0.91 | (0.90, 0.93) | 0.91 | (0.87, 0.95) | 0.99 | (0.98, 0.99) | 0.91 | (0.87, 0.95) | 0.99 | (0.98, 0.99) | 78.90 | (49.09, 126.82) | 0.09 | (0.06, 0.14) |
| nprp_picu_InptPedCap_ PICU_YN | aha_picu_pedicbd AND.aha_picu_pedichos | 0.91 | (0.90, 0.93) | 0.91 | (0.87, 0.95) | 0.99 | (0.98, 0.99) | 0.91 | (0.87, 0.95) | 0.99 | (0.98, 0.99) | 78.90 | (49.09, 126.82) | 0.09 | (0.06, 0.14) |
| nprp_picu_InptPedCap_ PICU_YN | aha_picu_pedichos | 0.88 | (0.87, 0.90) | 0.93 | (0.90, 0.97) | 0.98 | (0.97, 0.98) | 0.84 | (0.79, 0.89) | 0.99 | (0.99, 1.00) | 38.09 | (27.53, 52.70) | 0.07 | (0.04, 0.11) |
| nprp_picu_InptPedCap_ PICU_YN | aha_picu_pedicbd OR.aha_picu_pedichos | 0.88 | (0.87, 0.90) | 0.93 | (0.90, 0.97) | 0.98 | (0.97, 0.98) | 0.84 | (0.79, 0.89) | 0.99 | (0.99, 1.00) | 38.09 | (27.53, 52.70) | 0.07 | (0.04, 0.11) |
| nprp_picu_InptPedCap_ PICU_YN AND.nprp_picu_child_to_ adult | aha_picu_pedicbd | 0.51 | (0.49, 0.53) | 0.91 | (0.84, 0.97) | 0.92 | (0.91, 0.93) | 0.36 | (0.29, 0.42) | 1.00 | (0.99, 1.00) | 11.37 | (9.48, 13.62) | 0.10 | (0.05, 0.20) |
| nprp_picu_InptPedCap_ PICU_YN | aha_picu_pedicbd AND.aha_picu_pedichos | 0.51 | (0.49, 0.53) | 0.91 | (0.84, 0.97) | 0.92 | (0.91, 0.93) | 0.36 | (0.29, 0.42) | 1.00 | (0.99, 1.00) | 11.37 | (9.48, 13.62) | 0.10 | (0.05, 0.20) |

© 2025 McDaniel CE et al. *JAMA Network Open*.

| | | | | | | | | | | | | | | | |
|---|---|---|---|---|---|---|---|---|---|---|---|---|---|---|---|
| AND.nprp_picu_child_to_adult | | | | | | | | | | | | | | | |
| nprp_picu_InptPedCap_PICU_YN OR.nprp_picu_child_to_adult | aha_picu_pedichos | 0.49 | (0.46, 0.51) | 0.33 | (0.29, 0.37) | 0.98 | (0.97, 0.99) | 0.90 | (0.86, 0.94) | 0.73 | (0.70, 0.75) | 15.52 | (10.20, 23.63) | 0.68 | (0.64, 0.72) |
| nprp_picu_InptPedCap_PICU_YN OR.nprp_picu_child_to_adult | aha_picu_pedicbd OR.aha_picu_pedichos | 0.49 | (0.46, 0.51) | 0.33 | (0.29, 0.37) | 0.98 | (0.97, 0.99) | 0.90 | (0.86, 0.94) | 0.73 | (0.70, 0.75) | 15.52 | (10.20, 23.63) | 0.68 | (0.64, 0.72) |
| nprp_picu_InptPedCap_PICU_YN AND.nprp_picu_child_to_adult | aha_picu_pedichos | 0.48 | (0.46, 0.51) | 0.94 | (0.88, 0.99) | 0.91 | (0.89, 0.92) | 0.33 | (0.27, 0.39) | 1.00 | (0.99, 1.00) | 10.03 | (8.51, 11.82) | 0.07 | (0.03, 0.17) |
| nprp_picu_InptPedCap_PICU_YN AND.nprp_picu_child_to_adult | aha_picu_pedicbd OR.aha_picu_pedichos | 0.48 | (0.46, 0.51) | 0.94 | (0.88, 0.99) | 0.91 | (0.89, 0.92) | 0.33 | (0.27, 0.39) | 1.00 | (0.99, 1.00) | 10.03 | (8.51, 11.82) | 0.07 | (0.03, 0.17) |
| nprp_picu_InptPedCap_PICU_YN OR.nprp_picu_child_to_adult | aha_picu_pedicbd | 0.47 | (0.45, 0.50) | 0.31 | (0.28, 0.35) | 0.99 | (0.98, 1.00) | 0.94 | (0.91, 0.98) | 0.72 | (0.70, 0.75) | 30.65 | (16.82, 55.84) | 0.69 | (0.66, 0.73) |
| nprp_picu_InptPedCap_PICU_YN OR.nprp_picu_child_to_adult | aha_picu_pedicbd AND.aha_picu_pedichos | 0.47 | (0.45, 0.50) | 0.31 | (0.28, 0.35) | 0.99 | (0.98, 1.00) | 0.94 | (0.91, 0.98) | 0.72 | (0.70, 0.75) | 30.65 | (16.82, 55.84) | 0.69 | (0.66, 0.73) |
| nprp_picu_child_to_adult | aha_picu_pedichos | 0.25 | (0.22, 0.27) | 0.18 | (0.15, 0.21) | 0.89 | (0.87, 0.90) | 0.39 | (0.32, 0.45) | 0.73 | (0.71, 0.76) | 1.59 | (1.24, 2.04) | 0.92 | (0.88, 0.97) |
| nprp_picu_child_to_adult | aha_picu_pedicbd OR.aha_picu_pedichos | 0.25 | (0.22, 0.27) | 0.18 | (0.15, 0.21) | 0.89 | (0.87, 0.90) | 0.39 | (0.32, 0.45) | 0.73 | (0.71, 0.76) | 1.59 | (1.24, 2.04) | 0.92 | (0.88, 0.97) |
| nprp_picu_child_to_adult | aha_picu_pedicbd | 0.23 | (0.21, 0.25) | 0.16 | (0.13, 0.19) | 0.90 | (0.88, 0.92) | 0.39 | (0.32, 0.45) | 0.73 | (0.71, 0.75) | 1.59 | (1.22, 2.07) | 0.93 | (0.89, 0.98) |
| nprp_picu_child_to_adult | aha_picu_pedicbd AND.aha_picu_pedichos | 0.23 | (0.21, 0.25) | 0.16 | (0.13, 0.19) | 0.90 | (0.88, 0.92) | 0.39 | (0.32, 0.45) | 0.73 | (0.71, 0.75) | 1.59 | (1.22, 2.07) | 0.93 | (0.89, 0.98) |
| Pediatric Intensive Care: POS to NPRP | | | | | | | | | | | | | | | |
| nprp_picu_InptPedCap_PICU_YN | pos_picu | 0.62 | (0.60, 0.64) | 0.80 | (0.74, 0.85) | 0.90 | (0.88, 0.91) | 0.51 | (0.45, 0.56) | 0.97 | (0.96, 0.98) | 7.70 | (6.52, 9.09) | 0.23 | (0.17, 0.30) |
| nprp_picu_InptPedCap_PICU_YN OR.nprp_picu_child_to_adult | pos_picu | 0.47 | (0.45, 0.50) | 0.36 | (0.32, 0.40) | 0.91 | (0.89, 0.93) | 0.69 | (0.64, 0.74) | 0.72 | (0.70, 0.74) | 4.02 | (3.23, 5.01) | 0.70 | (0.66, 0.75) |
| nprp_picu_InptPedCap_PICU_YN AND.nprp_picu_child_to_adult | pos_picu | 0.32 | (0.29, 0.34) | 0.79 | (0.70, 0.88) | 0.84 | (0.83, 0.86) | 0.20 | (0.15, 0.24) | 0.99 | (0.98, 0.99) | 5.07 | (4.32, 5.96) | 0.25 | (0.16, 0.38) |
| nprp_picu_child_to_adult | pos_picu | 0.30 | (0.28, 0.32) | 0.25 | (0.21, 0.29) | 0.84 | (0.82, 0.86) | 0.38 | (0.32, 0.43) | 0.74 | (0.71, 0.76) | 1.54 | (1.26, 1.89) | 0.90 | (0.85, 0.95) |

© 2025 McDaniel CE et al. *JAMA Network Open*.

**eTable 6.** Exploratory Model Test Characteristics of a Combined Dataset of AHA and POS Using the NPRP as the Gold Standard for Provision of Pediatric Services

| N=3089 Hospitals | **Sensitivity** (95% CI) | **Specificity** (95% CI) | **PPV** (95% CI) | **NPV** (95% CI) | **LR +** (95% CI) | **LR-** (95% CI) |
|---|---|---|---|---|---|---|
| **Newborn care** | | | | | | |
| Rule-based | 0.98 (0.97 - 1.00) | 0.85 (0.81 - 0.90) | 0.90 (0.87 - 0.93) | 0.97 (0.95 - 0.99) | 6.73 (5.01 - 9.03) | 0.02 (0.01 - 0.04) |
| DTA-RF | 0.97 (0.95 - 0.99) | 0.87 (0.82 - 0.91) | 0.91 (0.88 - 0.94) | 0.96 (0.93 - 0.98) | 7.22 (5.30 – 9.83) | 0.03 (0.02 - 0.06) |
| DTA-XGBoost | 0.98 (0.97 - 1.00) | 0.86 (0.82 - 0.90) | 0.91 (0.88 - 0.94) | 0.97 (0.95 - 0.99) | 7.10 (5.24 - 9.62) | 0.02 (0.01 - 0.04) |
| Logistic Regression | 0.96 (0.93 - 0.98) | 0.86 (0.82 - 0.90) | 0.90 (0.87 - 0.93) | 0.93 (0.90 - 0.96) | 6.90 (5.09 - 9.35) | 0.05 (0.03 - 0.08) |
| **Neonatal Intensive Care** | | | | | | |
| Rule-based | 0.85 (0.79 - 0.90) | 0.94 (0.92 - 0.96) | 0.82 (0.77 - 0.88) | 0.95 (0.93 - 0.97) | 13.93 (9.67 - 20.07) | 0.16 (0.11 - 0.24) |
| DTA-RF | 0.81 (0.75 - 0.88) | 0.95 (0.94 - 0.97) | 0.86 (0.80 - 0.91)) | 0.94 (0.92 - 0.96) | 17.87 (11.69 - 27.32) | 0.19 (0.14 - 0.27) |
| DTA-XGBoost | 0.85 (0.79 - 0.90) | 0.95 (0.93 - 0.97) | 0.86 (0.80 - 0.91) | 0.95 (0.93 - 0.97) | 17.73 (11.73 - 26.80) | 0.16 (0.11 - 0.23) |
| Logistic Regression | 0.83 (0.77 - 0.89) | 0.95 (0.93 - 0.97) | 0.85 (0.79 - 0.91) | 0.94 (0.92 - 0.96) | 16.57 (11.06 - 24.84) | 0.18 (0.13 - 0.26) |
| **General Pediatric Inpatient Care** | | | | | | |
| Rule-based | 0.81 (0.78 - 0.85) | 0.47 (0.40 - 0.54) | 0.76 (0.73 - 0.80) | 0.54 (0.47 - 0.62) | 1.53 (1.34 - 1.76) | 0.40 (0.31 - 0.51) |
| DTA-RF | 0.91 (0.88 - 0.94) | 0.42 (0.35 - 0.49) | 0.77 (0.73 - 0.81) | 0.69 (0.61 - 0.77) | 1.57 (1.39 - 1.77) | 0.21 (0.15 - 0.30) |
| DTA-XGBoost | 0.85 (0.81 - 0.88) | 0.48 (0.42 - 0.55) | 0.78 (0.74 - 0.81) | 0.60 (0.52 - 0.68) | 1.64 (1.43 - 1.89) | 0.32 (0.24 - 0.41) |
| Logistic Regression | 0.85 (0.82 - 0.89) | 0.57 (0.50 - 0.64) | 0.81 (0.77 - 0.84) | 0.65 (0.57 - 0.72) | 1.98 (1.68 - 2.34) | 0.26 (0.20 - 0.34) |
| **Pediatric Intensive Care** | | | | | | |
| Rule-based | 0.90 (0.81 - 0.98) | 0.98 (0.97 - 0.99) | 0.79 (0.68 - 0.89) | 0.99 (0.98 - 1.00) | 42.50 (24.09 - 74.98) | 0.10 (0.05 - 0.24) |
| DTA-RF | 0.82 (0.71 - 0.92) | 0.99 (0.98 - 1.00) | 0.87 (0.77 - 0.97) | 0.98 (0.97 - 0.99) | 77.28 (34.48 - 173.18) | 0.19 (0.10 - 0.34) |
| DTA-XGBoost | 0.84 (0.73 - 0.94) | 0.99 (0.99 - 1.00) | 0.93 (0.86 - 1.01) | 0.99 (0.98 - 1.00) | 158.42 (50.90 - 493.06) | 0.16 (0.09 - 0.31) |
| Logistic Regression | 0.80 (0.68 - 0.91) | 1.00 (0.99 - 1.00) | 0.95 (0.89 - 1.02) | 0.98 (0.97 - 0.99) | 226.04 (56.26 - 908.19) | 0.20 (0.12 - 0.36) |

Abbreviations: AHA, American Health Association Annual Survey; DTA-RF, Decision Tree Analysis – Random Forest; DTA-XGBoost, Decision Tree Analysis – XGBoost; LR, Likelihood Ratio; NPV, Negative Predictive Value; POS, Center for Medicare and Medicaid Services Provider of Service File; PPV, Positive Predictive Value

© 2025 McDaniel CE et al. *JAMA Network Open*.
