# Supplementary material for: Identifying Inpatient Pediatric Services Across National Datasets
Source: JAMA Netw Open. 2025 Jun 3;8(6):e2513527. doi: 10.1001/jamanetworkopen.2025.13527 (PMC12134956; doi:10.1001/jamanetworkopen.2025.13527)
Supplement: Supplement 2. — Data Sharing Statement [file jamanetwopen-e2513527-s002.pdf]

Data Sharing Statement

Data
Data available: No

Additional Information
Explanation for why data not available: The AHA and NPRP datasets are proprietary. However, all of our analysis is available.
